# HiREX: High-Throughput Reactivity Exploration for Extended Databases of Transition-Metal Catalysts

**DOI:** 10.1021/acs.jcim.3c00660

**Published:** 2023-09-22

**Authors:** Ali Hashemi, Sana Bougueroua, Marie-Pierre Gaigeot, Evgeny A. Pidko

**Affiliations:** † Inorganic Systems Engineering, Department of Chemical Engineering, Faculty of Applied Sciences, Delft University of Technology, Van der Maasweg 9, Delft 2629 HZ, The Netherlands; ‡ Laboratoire Analyse et Modélisation pour la Biologie et l’Environnement (LAMBE) UMR8587, Paris-Saclay, Univ Evry, CY Cergy Paris Université, CNRS, LAMBE UMR8587, Evry-Courcouronnes 91025, France

## Abstract

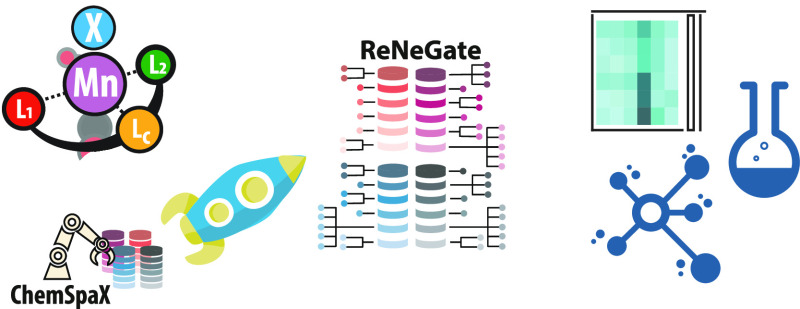

A method is introduced for the automated analysis of reactivity exploration for extended in silico databases of transition-metal catalysts. The proposed workflow is designed to tackle two key challenges for bias-free mechanistic explorations on large databases of catalysts: (1) automated exploration of the chemical space around each catalyst with unique structural and chemical features and (2) automated analysis of the resulting large chemical data sets. To address these challenges, we have extended the application of our previously developed ReNeGate method for bias-free reactivity exploration and implemented an automated analysis procedure to identify the classes of reactivity patterns within specific catalyst groups. Our procedure applied to an extended series of representative Mn(I) pincer complexes revealed correlations between structural and reactive features, pointing to new channels for catalyst transformation under the reaction conditions. Such an automated high-throughput virtual screening of systematically generated hypothetical catalyst data sets opens new opportunities for the design of high-performance catalysts as well as an accelerated method for expert bias-free high-throughput in silico reactivity exploration.

## 1 Introduction

The need to create molecular structures with tailored (bio)chemical functions has been driving chemical research. Traditional experimental chemistry, conventionally guided by intuition, chemical knowledge, and serendipity, has been so far successful in discovering functional molecular frameworks and improving their characteristics toward desired properties. For example, molecular catalysts are decorated with diverse functional groups to explore their activity and stability and devise possible strategies to improve performance.^1–3^ However, such an approach is always limited by the synthetic and physical availability of the particular chemicals to experimentalists, which substantially limits the scope of the exploration of the theoretically accessible catalysis space. While in vitro functionalization can provide insights into the chemical design principles behind high activity, selectivity, and stability, it can also be demanding in terms of time and resources. As a result, computational (in silico) molecular design is becoming a practical and promising alternative due to recent advancements in quantum chemical methods and high-performance computing.^1–10^ High-throughput computational methods can help create effective functionalization strategies by exploring geometries within the local chemical space of a given molecular framework.^4^

Organometallic chemistry space presented for development of new catalysts for useful chemical transformations is very large: it can be viewed as a combinatorics of the (i) transition metal (TM) centers with (ii) varied oxidation states in (iii) different coordination environments established by the organic ligands. The common practice is to assume that the variations in the chemistry of the ligand environment (ligand functionalization) do not affect the main mechanistic and reactivity properties but only the energetics of the associated paths. Therefore, one can first investigate in detail the mechanism and reactivity of a particular selected catalyst, followed by high-throughput screening using the descriptors or targets identified for this specific complex. However, one can expect that the chemical modification of a catalyst can open up new mechanistic possibilities. The catalytic properties of the organometallic complexes are governed by a much wider reactivity space. Therefore, to enable the high-throughput in silico catalyst screening, one ideally has to explore the reactivity of each member of the catalyst library, with the associated problem of the combinatorial explosion resulting in an extremely large and complex data sets of results that need to be analyzed. Furthermore, the featurization and labeling of homogeneous catalysts based on their structural features as well as distinct reactivities remain a challenge when exploring unexpected chemistries. In the context of expert-bias-free reactivity exploration, methods for correlating structural and reactive features and extraction of reaction classes would be required. Experimental investigations of finding catalysts with optimal properties for defined functions are limited to a few accessible variants of the functionalized ligand scaffold and ligand combinations. Theoretical investigations, on the other hand, can be much broader by design and can navigate through arbitrary regions of the chemical space in an exploratory search for defined properties. Such a chemical space exploration can be guided by systematic functionalization of scaffolds to find highly stable and active catalysts. Modern computational chemistry methods are instrumental for such a task and have been used successfully in the past to screen through large databases of functionalized TM complexes^11,12^ for activity, regioselectivity, and ligand effects.^13–15^ In our studies aiming at understanding the design principles for homogeneous hydrogenation catalysis by manganese,^16,17^ Krieger et al. carried out a high-throughput thermodynamic analysis of a virtual library of Mn pincer catalysts within the constraints of predefined deactivation chemistry.^18^

**Figure 1:**
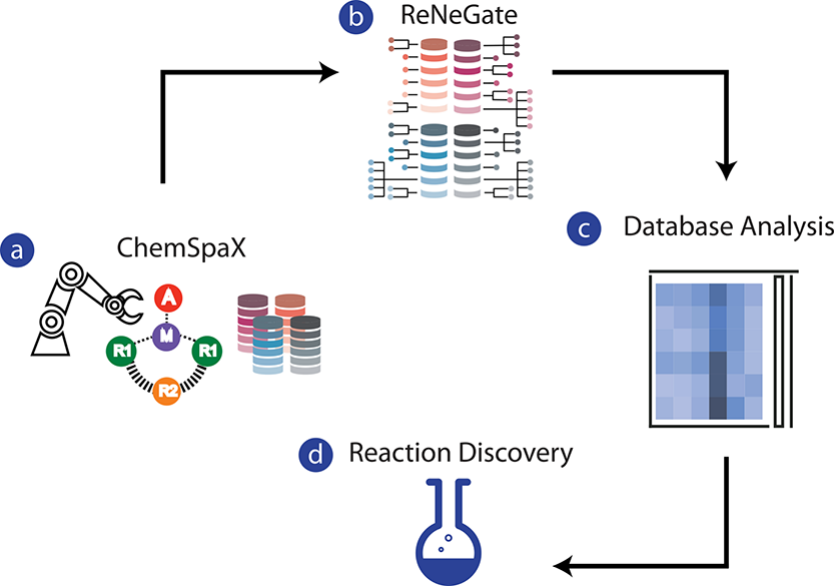
Computational workflow for (a) generation, (b) chemical space exploration, and (c) analysis of extended in silico catalyst data sets for (d) the discovery of new reactivities and particularly those giving rise to the thermodynamically driven catalyst degradation/deactivation.

Here, we aim at removing expert bias from the reactivity analysis by integrating the automated procedures for organometallic complex generation, reactivity exploration, and analysis into the unified HiREX workflow (Figure 1). The ChemSpaX^19^ fully automated procedure is used for ligand functionalization and generation of in silico catalyst library that forms the input for the dynamic reactivity explorations with the ReNeGate^20^ procedure. Similar to the original implementation^20^, RMSD biased metadynamics simulations as implemented in CREST^21^ have been used to exhaustively explore the chemical space around each catalyst. The reactivity exploration results are organized in a database with structural and reactive properties observed for every record as features in the database. The database is then analyzed to find correlations between structural and reactive features, followed by the extraction of the reaction classes identified for each catalyst entry and family. Such systematic, automated, and bias-free exploration and analysis has provided insights in defining reaction classes correlated with specific combinations of backbone and ligand modifications. It should be noted that we operate within the assumption that the computational methodologies used for chemical space exploration and electronic structure calculations (e.g., DFT functionals) are sufficiently accurate for the given chemical problem. Thus, our workflow inherits the inaccuracies derived from the description of the model system using a specific level of theory. However, we have designed our workflow to be able to analyze trajectories of arbitrary accuracy and complexity and are agnostic in our analysis to any specific level of accuracy.^20^

The article is organized as follows: first, we provide an extensive description of the methodological aspects of the presented workflow. We start with the introduction of the functionalization strategy ChemSpaX used to construct the extended synthetic pincer catalyst set, followed by the description of the automated dynamic exploration workflow ReNeGate applied to this data set. The methodology section is concluded by the presentation of the automated analysis method applied to multiple reactive trajectories generated for the extended synthetic catalyst data set. The results and discussion section present the application of this workflow on the selected families of Mn pincer catalysts. The paper is completed with a conclusion section. *HiREX* code is publicly available at: https://github.com/ahashemiche/HiREX.

## 2 Methods and Models

### 2.1 Mn Catalyst Library: ChemSpaX Functionalization

In silico catalyst screening aims at analyzing the effect of the functionalization type and site (backbone/donor) on the reactivity behavior. We demonstrate the capabilities of the workflow by considering a specific class of Mn pincer homogeneous catalysts.^22–25^ The applicability of the presented methodology is, however, not limited to any specific class of compounds. Recently, Krieger et al. presented a computational study on a virtual library of Mn pincer catalysts within the constraints of the predefined mechanistic assumptions.^18^ Herein, we expand on that work by implementing the HiREX workflow and explore if unexpected reactivities can be discovered upon reduction of the expert bias.

**Figure 2:**
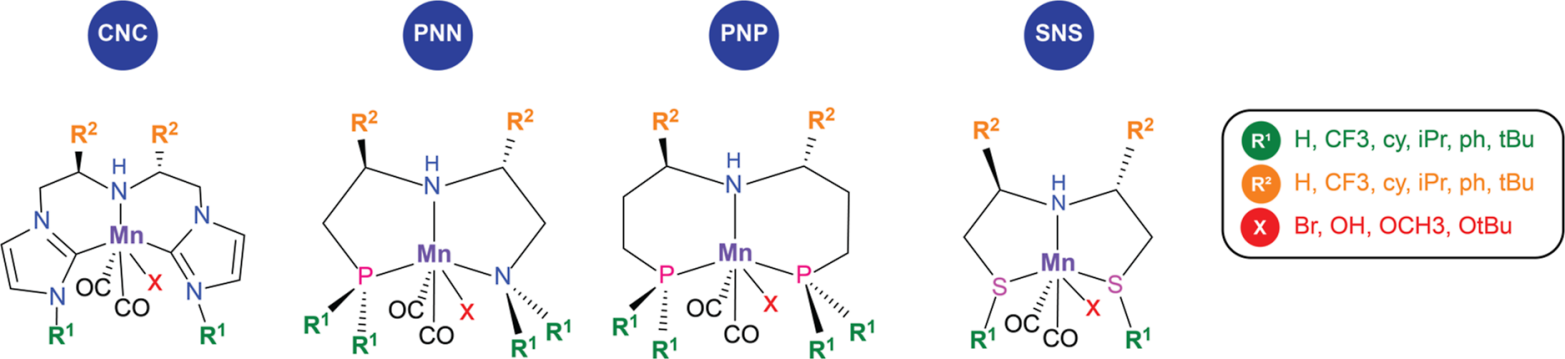
Representative 3d transition-metal (Mn) pincer scaffolds with the ligand modification R^1^, R^2^, and TM adduct (X) within the scope of this work. The functionalizations R^1^ and R^2^ include proton (H), trifluoromethyl (CF_3_), cyclohexyl (cy), isopropyl (^*i*^Pr), phenyl (Ph), and *tert*-butyl (^*t*^Bu); and adducts (X) can be represented by bromide (Br), hydroxyl (OH), methoxide (OCH_3_), and *tert*-butoxide (O^*t*^Bu) anions.

The ChemSpaX virtual Mn catalyst library used for this study contained complexes with four representative pincer scaffolds, namely, PNP, SNS, CNC, and PNN backbones coordinated to a Mn(I) center stabilized by CO ligands and an anionic (X) group (Figure 2).^18^

The design of the virtual library is guided by our focus on the application of Mn(I)-pincers^26–29^ in the context of hydrogenation catalysis for sustainable energy and chemistry applications.^30–36^ The ligands are representative of the choices made by experimentalists for screening purposes and cover a broad range of steric and electronic properties. Ligand modifications are carried out by varying functionalizations at R^1^ and R^2^ positions, which allow probing the effects of the first (donor atom directly coordinated with the Mn center) and second (backbone functionalization) coordination spheres, respectively. To simplify analysis, the library was limited to symmetric functionalizations for each site group, although this does not represent the fundamental limitation of the ChemSpaX procedure, which is described in detail elsewhere.^19^

For each pincer ligand, four different Mn-adducts were considered with Br^–^, OH^–^, OMe^–^, and O^*t*^Bu^–^ anions as the anionic ligands representing the precatalyst and/or intermediates (Figure 2). Among the set of X adducts, Br is a common precursor to the active form of manganese pincer catalysts.^36^ Such precursors often go through activation with a strong base to produce a highly active five coordinated complex. OR-adducts (OH, OCH_3_, and O^*t*^Bu) are usually formed upon addition of alcohol/water/base via metal ligand cooperative addition.^37,38^ Formation of such adducts may lead to temporary inhibition or even deactivation of the catalyst depending on their specific stability and reactivity.^29,39–41^

The different combinations of R^1^, R^2^, and X functionalization with the four pincer scaffolds gave rise to a virtual library containing 576 complexes that were further used for the reactivity exploration and analysis. Pincer complexes based on these ligands have been reported for various transition metals. Very efficient catalyst systems for a wide range of catalytic transformations have been established using 4d and 5d transition metal pincer complexes.^42–44^ Their 3d transition-metal counterparts often have lower catalytic efficiencies due to the tendency of such systems to deactivate under the reaction conditions, limiting their catalytic performance.^22,45^

### 2.2 Reactivity Explorations—the ReNeGate Workflow

The virtual precatalyst library was fed as the input for the dynamic mechanistic explorations. In order to remove expert bias from mechanistic studies and to discover new chemistries, our automated graph-theoretical methodology implemented in the ReNeGate^20^ workflow is used to explore the potential energy surface around each starting structure. In line with the design of HiREX workflow, we make no prior chemical assumptions and aim specifically at exploring if the variation in the nature of the primary and the auxiliary ligands influences the type of chemistry our automated workflow discovers.

The individual entities in the database are automatically provided in parallel as input to the exploration step, where the chemical space around each structure is exhaustively explored for alternative chemical structures and new reactions. The detailed description of the ReNeGate workflow is provided elsewhere.^20^ Further details on relevant molecular graph theory terminology and molecular graph theory in reaction identification are included in Section S1 of the Supporting Information. We briefly summarize the key elements of the procedure below.

The procedure involves an exhaustive reaction space exploration through root-mean-square deviation (RMSD) biased meta-dynamics at the semiempirical xTB level of theory using the CREST functionality in the GFN-xTB code.^21^ Recent studies demonstrate the sufficient accuracy of the xTB for high-throughput screening of TM complexes.^46^ Implementation of the RMSD bias in the metadynamics simulations helps with the exhaustiveness of the exploration. CREST workflow sets a penalty on the configurations that have already been visited by calculating relative structural RMSD values for new configurations. Reactive trajectories from all metadynamics runs for all input structures from the ChemSpaX library are provided in parallel with the ReNeGate graph-theoretical analysis tool to identify unique structures and compare the observed configurations with the respective reference structures (Section 2.3). Alternative explored structures for all starting geometries in the database are then collected in a global database. Each element in the database is labeled based on the known structural features of the reference structure (backbone scaffold, ligands at R^1^ and R^2^ position, adduct) as well as the calculated features including relative energies (Δ*E*, kcal mol^–1^) and specific reactivity observed for species compared to the starting reference structure (broken or formed bonds). The database is initially analyzed for correlations between structural features and observed reactivities.

### 2.3 Comparison with Reference Structures

Exploration results for each starting structure are analyzed to provide insights into possible reaction (or deactivation) channels for given catalyst structures.

**Figure 3:**
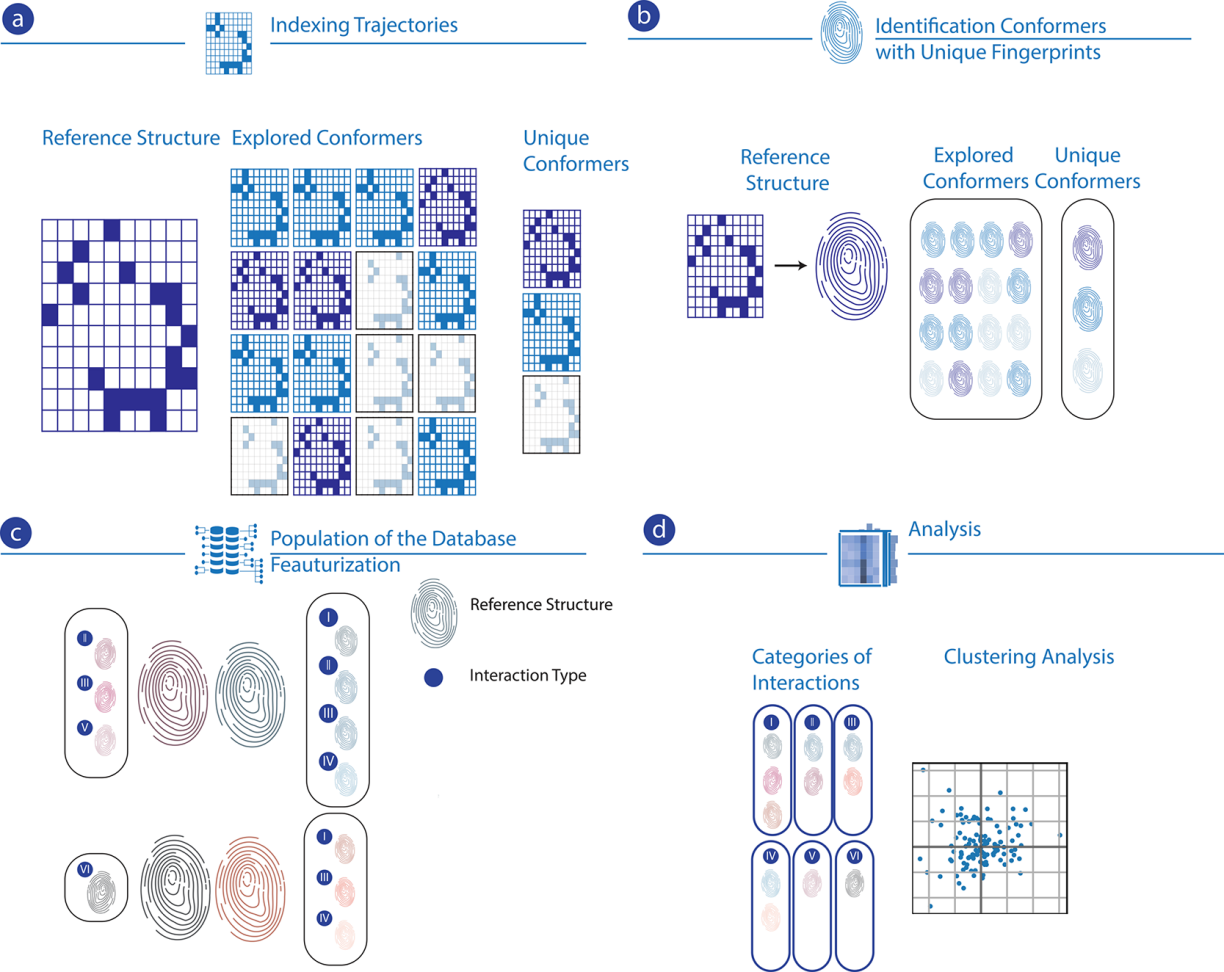
Pipeline for populating the reactivity databases of chemical structure based on comparisons with reference structures involving (a) indexing trajectories, (b) identification of conformers with unique fingerprints, (c) population of the database, and (d) analysis through categorization and clustering.

Compared to the protocol used in the original ReNeGate^20^ workflow, uniquely identified structures are compared against the respective reference structure, which allows the transfer of the extracted reaction labels between different backbone, R^1^, R^2^, and adduct classes. This provides global insights into the role of individual features in the energetics and reactivity patterns. The pipeline for populating databases of reactivities for chemical structures is illustrated in Figure 3. Indexing trajectories, identification of conformers with unique fingerprints, population of database, featurization, and analysis are the key steps for extracting insights from high-throughput virtual screenings of the current study. The algorithm proposed for making the comparison is discussed below.

Given a sequence of structures S_1_, S_2_, S_3_, ..., S_*n*_ where S_*i*_ represents configurations observed based on conformer exploration and S_1_ is the reference structure, the algorithm first analyzes the conformer changes based on the dynamics of interactions and bonds by applying isomorphism tests. Once the conformers are identified (noted C_1_, C_2_, ..., C_*m*_), we compare these conformers to the reference structure represented by the first conformer C_1_.

The algorithm includes the following steps:

1. Read the XYZ file, at each step:
  a. Construct the mixed graph^47^
  b. Extract the energy values from the trajectory analyzed at the desired level of theory
  c. Apply the isomorphism test with previously identified nonisomorphic conformers
  d. Identify conformers that are not isomorphic
  e. Compare bonding pattern between the new conformer and reference structure
  f. Identify the minimum and maximum value of energy for each conformer
2. Construct the graph of evolution of structures

The bonding patterns of the identified conformer and the respective reference are compared. For the bonds present in the new conformer, but not in the reference, we consider that the bond is formed (marked with a “+”) and, respectively, for the bonds present in the reference and not in the new conformer, the bond is considered broken (and marked with a “–”). Based on the changes in the bonding patterns, the differences between each conformer and the reference structure are stored in the database. In addition, we extract the energy values from the input file (the XYZ file that contains the structures S_1_, S_2_, S_3_, ..., S_*n*_) and calculate the relative energy difference of the conformer through comparison with the reference. For the purposes of this study, we consider the minimum energy value from the isomers’ list as the representative energy value assigned to the unique configuration for further analysis on the database. We then construct the graph of evolution of the reference structures (see Section S2 of the Supporting Information for a sample reaction network analysis). The vertices in the graph represent the identified conformers, and the edges connect the conformers and the reference structure. The representation includes the 2D image of the conformer, the list of structures that belong to this conformer, and the energy values extracted from the trajectory at the analysis level of theory, in the current implementation, either xTB or DFT. Similar to the original ReNeGate implementation, the reaction network calculations are carried out at two different levels of theory. Initial dynamic explorations are carried out at the GFN2-xTB^21^ level of theory, and then, the identified unique conformer structures are refined at B3LYP-D3/6-31g(d,p) level of theory with a GD3BJ dispersion correction^48^ and an implicit SMD model^49^ with the standard parameters for the THF solvent.

## 3 Results and Discussion

### 3.1 High-Throughput Reactivity Exploration

**Figure 4:**
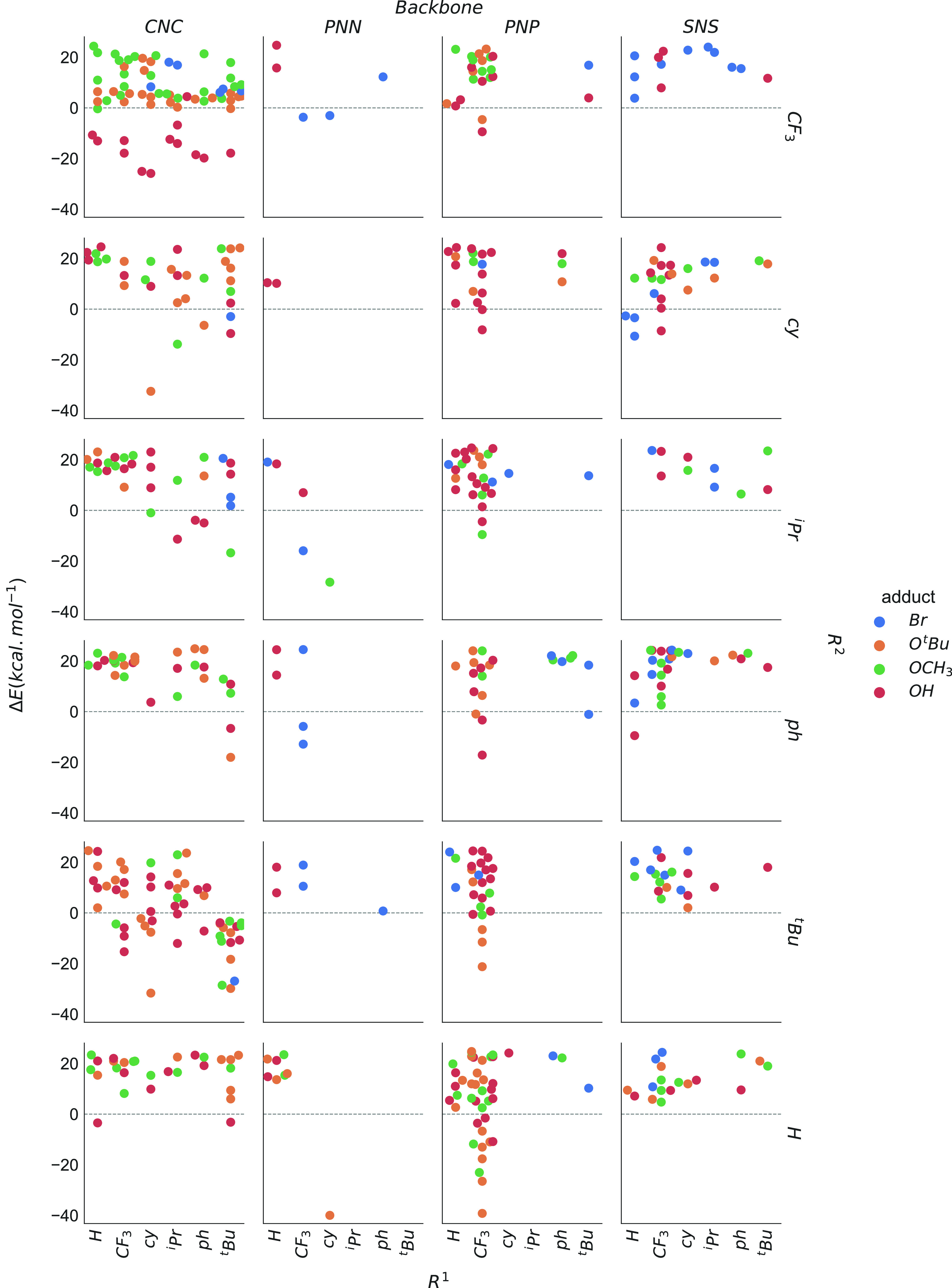
Exploration results for the evolution of the Mn–CNC, PNN, PNP, and SNS precatalysts in [−40, 25] kcal mol^–1^ stability range. The configurations are analyzed based on the backbone type and moieties at R^1^ and R^2^ positions. Data points are colored according to adduct X at the Mn center.

The primary results of the application of our automated reactivity analysis procedure to the virtual library of Mn pincer complexes are summarized in Figure S8 and demonstrate that the explored structures vary in terms of relative energy in the [−600, +100] kcal mol^–1^ range. Few species have been observed with relative energies as low as −600 kcal mol^–1^. Such observations mean that the starting structure in which we are interested will undergo extremely thermodynamically favorable configurational changes that render it unstable and, probably, very difficult to obtain experimentally. Therefore, we have limited further analysis in classification of reaction classes to species observed in the [−40, +25] kcal mol^–1^ range. The refined results in terms of the relative stabilities of the discovered species for each catalyst class/group are summarized in Figure 4. The introduced energy constraint is expected to improve the reliability of the defined reaction classes. This will also help to make sure that low energies obtained for some states are not artifacts coming from the automated design of structures.

Our results (Figure 4) highlight varied trends in reactivity as a function of pincer scaffold and functionalization. For example, the data in Figure 4 suggest that pincers featuring a CNC backbone can isomerize into more stable alternative species for all R^1^ and R^2^ functionalization and Mn–X adduct types. On the other hand, for the PNP family, the favorable transformation to stable alternative species is mainly limited to complexes with a CF_3_ substituent at R^1^. Systems with the PNN backbone are the least likely to form alternative configurations. These apparent trends can be readily deduced from the current representations of the stability diagrams. However, a deeper correlation analysis is necessary to gain more insights into the observed variations.

### 3.2 Correlation Analysis of the Structural Features and Reactivity

**Figure 5:**
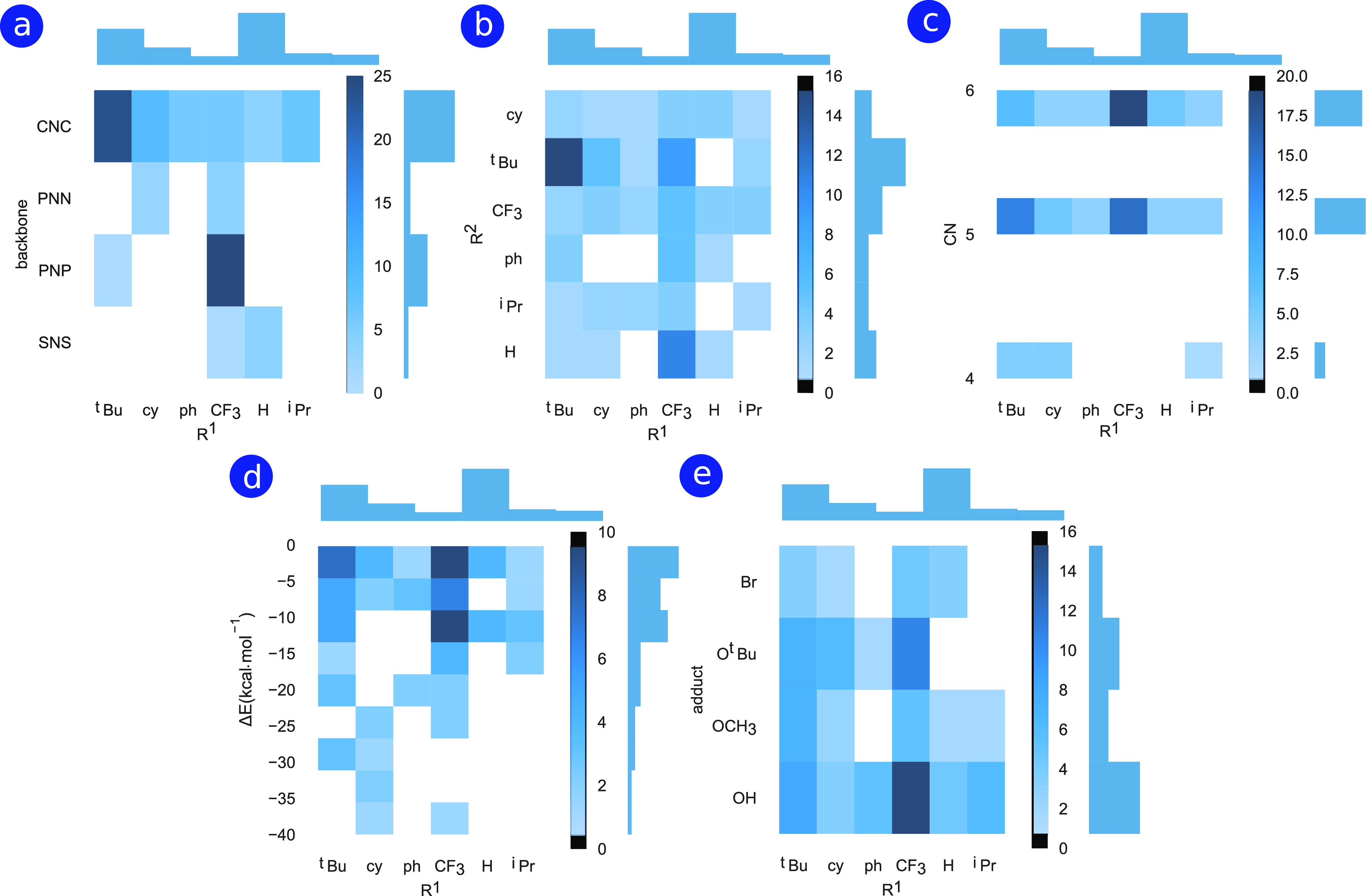
Histogram plots for the frequency of appearance of alternative stable species based on modified structures for CNC, PNP, PNN, and SNS catalysts (lower energies than the respective reference structure) as a function of feature combination (a) R^1^–backbone, (b) R^1^–R^2^, (c) R^1^–CN (coordination number of the metal center), and (d) R^1^–Δ*E* (relative energy), (e) R^1^–X.

Histogram plots in Figure 5 were made to illustrate how the combinations of different structural features are associated with the possibility of the formation of alternative stable configurations from a given precatalyst. These plots summarize the frequency of appearance of species with relative stabilities in the [0, −40] kcal mol^–1^ with respect to the given structural/compositional features. Figure 5 presents correlations between the R^1^ groups and other structural features including (a) catalyst backbone, (b) R^2^ groups, (c) coordination number (CN) of the metal (Mn), as well as (d) the energetics of the alternative species (Δ*E*) and (e) the adduct (X) type. The number of explored species in the database having specific features with different combinations of R^1^ with other parameters (backbone, R^2^, CN, Δ*E*, and X) is counted on the upper and right sides of each histogram. Specific combination cells are colored according to the frequency of the observations, with the color coding shown next to each plot. The frequency of observations of identified structures with specific feature combinations has been considered as a measure of correlation between structural and reactive features. A similar correlation analysis for configurations with energies within +25 kcal mol^–1^ from their respective reference state is collected in Figure S5 of the Supporting Information.

Figure 5a reveals a correlation between the presence of ^*t*^Bu and CF_3_ groups at the R^1^ positions for the CNC and PNP backbones. As opposed to catalysts with PNN, PNP, and SNS backbones, stable species with CNC backbones have been found with all variations of ligands at the R^1^ positions. Figure 5a shows that explorations on CNC catalysts have resulted in the formation of alternative stable species for all ligand substituents at the R^1^ positions. The CF_3_ group at R^1^ leads to the formation of more stable structures for PNN, PNP, and SNS backbones. Regarding the R^1^–R^2^ combinations (Figure 5b), the presence of ^*t*^Bu and CF_3_ at R^1^ always leads to the formation of alternative structures, indicating the direct involvement of R^1^ groups in the formation of stable intermediates.

The formation of stable alternative species with 5- and 6-coordinated Mn centers is, respectively, correlated with the presence of ^*t*^Bu and CF_3_ moieties at R^1^ (Figure 5c). 4-Coordinated Mn complexes were observed only for the pincer complexes with most bulky ^*t*^Bu, cy, and ^*i*^Pr substituents at the donor atoms. Interestingly, the R^1^ substitutions with the bulky ^*t*^Bu and cy as well as the most reactive CF_3_ groups are found to be correlated with the more pronounced stabilization of the alternative configurations (R^1^–Δ*E* correlations in Figure 5d). The analysis of the R^1^–X correlations presented in Figure 5e suggests that the OH adducts universally tend to convert to other more stable configurations for all of the R^1^ functionalizations. This emphasizes the role of OH ligand in forming stable structures, and this will be discussed in detail below. The diversity of the species explored based on the type of the adduct follows the order OH > O^*t*^Bu > OCH_3_ > Br. Energetics of the observed structures colored based on different adducts are further illustrated in Figures S6–S8 for [−40, 0], [−40, 25], and [−600, 100] kcal mol^–1^ ranges, respectively. These insights from correlations observed between the structural and reactive features of the catalysts provide clues for a more detailed analysis of the observed species and new chemistries, as shown in Section 4.

### 3.3 Finding the Most Frequent Classes of Interactions: K-Mode Clustering

In the next step toward implementing data-driven analysis of large databases of homogeneous catalyst structures, we have leveraged on labeled data organized based on automated exploration of the chemical space of the complete catalyst library. We have enumerated the global distinct types of interactions leading to alternative stable species and subsequently performed clustering to find the most frequent modes. Clustering, in general, is an unsupervised learning method whose task is to divide the population or data points into a certain number of groups such that data points belonging to the same group are similar to each other and dissimilar to the data points in the other groups. It is basically a collection of objects based on their similarity and dissimilarity between them. K-mode clustering^50^ is one of the unsupervised machine learning algorithms that is used to cluster categorical variables. Here, we used the kmode 0.12.2^50^ library for categorical clustering based on the reactivities of the observed species.

**Figure 6:**
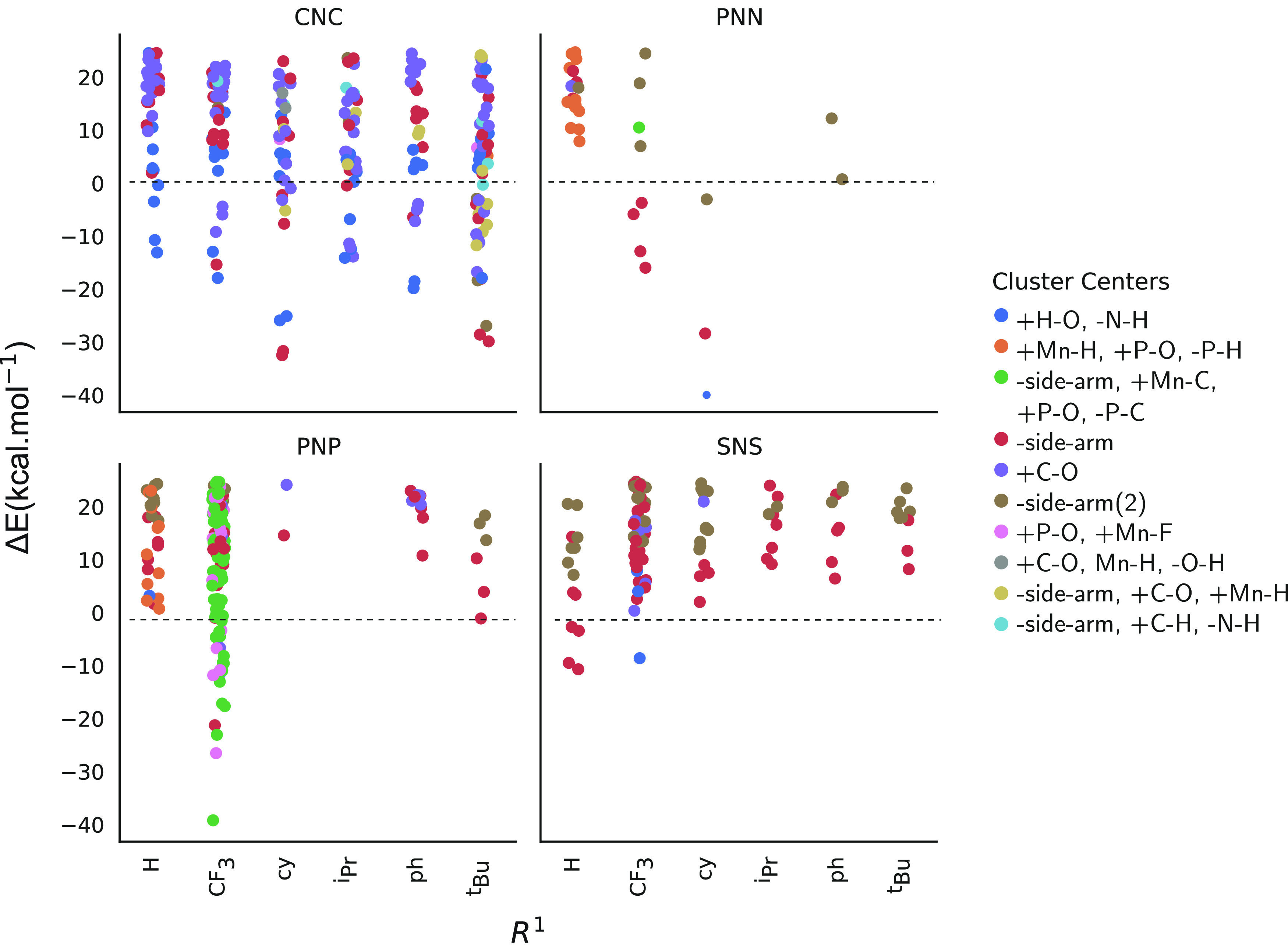
Top 10 clusters representing the most frequent types of interactions observed for different R^1^, R^2^ ligand, and backbone combinations. The energies for species explored in the [−40, +25 kcal mol^–1^] energy window are plotted as a function of ligand modification at the R^1^ position. Data points are colored by the type of interactions defined by the cluster centers (10 centers).

The results of the clustering analysis are summarized in Figure 6 by presenting the relative energies of the alternative configurations discovered for each of the backbone types and classified according to the interaction type realized in them. Analyzing the nature of interactions identified in Figure 6, we can draw insights into the new reactivity of Mn(I) pincers and their correlations with the type of backbone and modifications to the ligand backbones at the R^1^ and R^2^ positions. Filtering the new transformations in the database of events has led to three different sets of reactions:

- Decoordination of the Mn–D (D = C, N, and P) bonds
- Nucleophilic attack on the carbonyl ligands
- Migrations of the CF_3_ moieties

To the best of our knowledge, such reactivities have not been discussed before in the context of hydrogenation catalysis by Mn(I) pincer systems. However, this is not fully unprecedented. Indeed, earlier studies reported similar reactivities as those mentioned above for different organometallic and homogeneous catalyst systems, which are discussed in more detail in Section 4.

The cluster centers identified and used to classify species in Figure 6 are explained in terms of the chemistry they represent:

- **+H–O** and **–N–H**: for catalysts with CNC backbone and OH (or OCH_3_) adducts with R^2^=CF_3_, the hydrogen atom on the backbone nitrogen can migrate to the adduct and further be followed by the detachment of the adduct from the Mn center (Table S3).
- **+Mn–H, +P–O,** and **–P–H**: for catalyst with PNN and PNP backbones with R^1^=H, manganese hydride species is formed via the transfer of H from R^1^ to Mn (+Mn–H, −P–H) followed by bridging of the alkoxide species between Mn and the phosphorus atom (+P–O).
**-side arm, +Mn–C, +P–O,** and **–P–C**: for catalysts with the PNP backbone (also a single case for the PNN catalyst), the side arm (-side arm) P-donor decoordinates from the Mn center, and the CF_3_ moiety migrates from R^1^ to Mn (+Mn–C, +P–O, and −P–C).
**-side arm(−Mn–N, −Mn–C, −Mn–P,** and **–Mn–S)**: decoordination of the side arm observed for the catalyst with CNC, PNN, PNP, and SNS backbones resulting in new stable configurations.
- **“+C–O”, “+C–O, +Mn–H, −O–H”, “-side arm, + C–O,** and **+ Mn–H”**: for catalysts with CNC backbone, nucleophilic attack from adducts (+C–O) into carbonyls is observed for all R^1^ variations. Such interactions have been observed to be accompanied by agostic interactions (+Mn–H) or the dissociation of the side arm.
**-side arm(2)**: decoordination of two side arms observed mostly for catalysts with the SNS backbone with all types of R^1^ substitution. Such reactivity is also observed for catalysts with PNN backbone with R^1^=CF_3_ and for PNP backbones with R^1^=H or ^*t*^Bu.
- **+P–O** and **+Mn–F**: for PNP catalysts with R^1^=CF_3_, the alkoxide adduct (X = OR) is transferred into the bridging position between Mn and P (+P–O), the new state is stabilized by an agostic Mn–F interactions.
**-side arm, + C–H,** and **–N–H**: decoordination of the side arm accompanied by the insertion of the hydride from the backbone amine has been observed for catalysts with CNC backbone when either bulky (^*i*^Pr or ^*t*^Bu) or CF_3_ moieties are present at R^1^ position.

Analysis based on the nature of the bond changes shows that cleavage of the Mn donor bonds is most frequently observed and is accompanied by other reactive events. As shown in Figure 6, the decoordination of the donor ligands allows the formation of stable structures for catalysts with CNC, PNN, PNP, and SNS backbones, opening thus paths toward new and unexpected chemistries, which are discussed in more details in the next section.

## 4 New Chemistry

### 4.1 Decoordination of the Mn–D (D = C, N, and P) Bonds

In coordination chemistry and catalysis, the reactivity and stability of catalytic species can be tuned through ligand hemilability.^51^ The partial and reversible displacement of the ligand may provide additional kinetic stabilization of the reactive catalytic complexes and serve as the point of entry into the catalytic cycle or facilitate the regeneration of the catalytically active species at the stage of product release. The facile interconversion between the fully and hemi-coordinated states may be critical for the fast catalytic turnover rate.^52^ On the other hand, the increased reactivity of the complex due to the (partial) ligand dissociation may give rise to further conversion resulting in the long-term catalyst deactivation. We therefore first focused the more in-depth reactivity analysis on the phenomenon of labile donor atoms of the pincer scaffold. The reactivity patterns of structures were queried for the decoordination of either or a combination of side arm donor ligands (−Mn–C, −Mn–N, −Mn–P, or −Mn–S). Presence of one or more of these interactions for a structure has then been marked as an indicator of the probable presence of hemilabile Mn-donor bonds in the reference structures. Figure S16 summarizes the results of the automated hemilability detection by showing the relative stabilities of the different structures in the database labeled as having the –Mn–D (D = C, N, P, and S) feature indicating hemilability.

Our analysis reveals that the decoordination of Mn–N and Mn–S bonds are the most frequent side arm decoordination events (Figure S16, Table S7). For clarity, we have clustered structures including Mn–N decoordination with a similar “–Mn–N Set” label. Further analysis on the different R^1^–R^2^-adduct combinations leading to specific reactivities including −Mn–N has been summarized in Section S3 of the Supporting Information. Phosphine dissociation in PNN and PNP catalyst families is rare, with most cases found for the bulky R^1^=Ph, cy, and ^*t*^Bu. The detailed plots including expanded lability interactions are included in the Supporting Information and will be discussed here.

For CNC catalysts, bulky ^*t*^Bu and cy groups at the backbone R^2^ sites favor the rather unexpected decoordination of the central amino donor (−Mn–N) of the pincer ligand. A similar behavior (−Mn–N) is also observed when CF_3_ or no functional groups are present at the R^2^ position along with H or CF_3_ groups at R^1^. Ligand dissociation was not observed for all configurations of the Br adduct of Mn–CNC (Figure S11). For PNN complexes, the presence of electron-withdrawing CF_3_ groups at either of R^1^ or R^2^ position generally promotes the N-donor hemilability (−Mn–N) that results in a substantial stabilization (by −200 kcal mol^–1^) of the Mn complexes for all R^1^–R^2^–X Mn–PNN combinations. Decoordination of both Mn–N bonds “–Mn–N(2)”, “–Mn–N, −Mn–P” or complete decoordination of the ligands is uniquely observed for the species with R^1^=CF_3_ (Figure S12).

The decoordination behavior of Mn-PNP catalysts includes the dissociation of both the central amino (−Mn–N) and phosphine side arm (−Mn–P) donors. While decoordination of the Mn–N bond is observed for all R^1^–R^2^ combinations, decoordination of one Mn–P is observed only for R^1^=H or CF_3_. Furthermore, complete dissociation of all donor atoms of the backbone is observed for R^1^=CF_3_ and R^2^=H (Figure S13). For the catalysts with the SNS pincer scaffolds, the ligand lability was also detected for all donor atoms of the pincer [−Mn–N, −Mn–S, and −Mn–S(2), where the latter indicates the decoordination of both S donor atoms]. The presence of CF_3_ groups at R^1^ again promotes the dissociation of both the N and S donors. In this catalyst family, the dissociation of the central Mn–N bond is less common than the cleavage of the weaker Mn–S bonds, and it was observed for the R^1^=CF_3_ and R=cy combination (Figure S14). In order to identify possible hemilabile states and to distinguish between the side arm ligand decoordination and hemilability, we further classified the decoordination reactivities to only events, leading to more stable structures compared to the reference structure (Figure S16). Results in Figures S11 and S15 reveal that bulky (^*t*^Bu and cy) groups at R^1^ position of CNC induce decoordination of the central N donor (−Mn–N) and may also promote the dissociation of the NHC side arms (−Mn–C). Decoordination of the NHC moieties of CNC (−Mn–C bond) is also observed with the ^*t*^Bu-cy combination of the R^1^–R^2^ groups. Presence of CF_3_ on the phosphine donors of the PNP backbone also promotes the cleavage of the side arm (Mn–P or Mn–N) ligands, whereas in the case of the SNS pincers, the H atoms at R^1^ sites generally promote the cleavage of one or both of the Mn–S side arm donor ligands (Figures S13 and S14). Most frequently, the energetically favorable dissociation of the central nitrogen donor of the pincer scaffold (−Mn–N bond) has been observed for CNC, PNN, or PNP backbones. The dissociation of the Mn–C bond (either as “–Mn–C” or “–Mn–N, −Mn–C”) is unique for the CNC backbone. Other interactions leading to the complete dissociation of the backbone ligands, including [“(−Mn–N)_2_” for PNN backbones, “(−Mn–N)_2_, −Mn–P” for catalysts with the PNP backbone, “–Mn–N, (−Mn–S)2” for catalysts with the SNS backbone] have been also observed, but they led to structures with generally higher energies than those where only a single side arm has dissociated. Presence of ^*t*^Bu groups at the R^2^ position of the backbone promotes the hemilability of the side arm donor atoms for all catalysts. In the meantime, for PNN, PNP, and SNS catalysts, the favorable dissociation of the side arm is not affected by the R^2^ functionalization of the backbone but is solely controlled by the nature of the R^1^ group at the donor moiety. The diversity of the hemilabile species explored based on the type of the adduct follows the order OH > O^*t*^Bu > OCH_3_ > Br.

### 4.2 Nucleophilic Attack on Carbonyl Ligands

**Figure 7:**
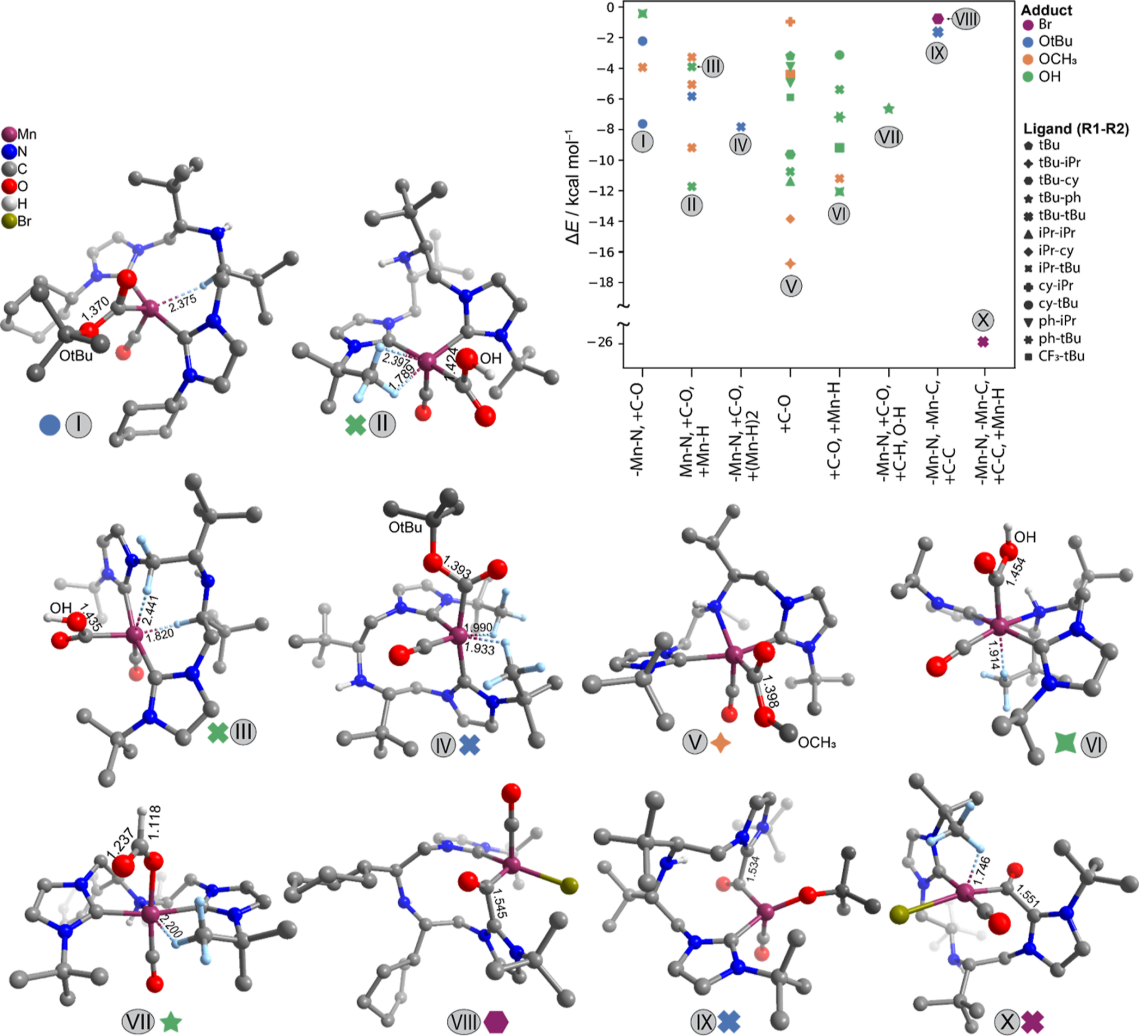
Optimized structures of the most stable species explored for CNC family with different R^1^–R^2^ combinations (hydrogen atoms are omitted for clarity, only the H atoms involved in agostic interactions with the Mn center are shown as light blue spheres). Schematic representations of the most stable structures are presented in Figure S9 of the Supporting Information

Nucleophilic attack and insertion into the carbonyls have been observed for all of the basic adducts (OH, OCH_3_, and O^*t*^Bu) in the database. This is in line with our previous hypothesis on the potential relevance of such a reactivity for the base-assisted deactivation paths of Mn catalysts stabilized by carbonyl CO ligands.^20,53,54^ Let us consider in more detail the revealed reactivity patterns by focusing on the Mn–CNC complex family. Figure 7 summarizes the results of the automated analysis and shows the optimized structures of the most stable species identified for each class (denoted with roman numerals in Figure 7). The energy values for the observed structures are plotted on the *Y*-axis against the set of reactivities observed for catalyst structures with the CNC backbone shown on the *X*-axis. Symbols representing modifications at the R^1^ and R^2^ positions, as well as color coding for adduct present on the Mn center, are shown in the legend.

Besides the migratory insertion of the basic hydroxide or alkoxide adduct, the NHC moieties of the CNC pincer have also been observed to attack the carbonyl ligands, as illustrated with structures VII, IX, and X (Figure 7). It should be noted that for the very bulky substituents where ^*t*^Bu and cy groups are, respectively, present at R^1^ and R^2^ positions, the central nitrogen ligand can also decoordinate, as observed in structure VII. Such transformations are accompanied by the dissociation of the amine donor of the pincer ligand, and the associated energy losses are compensated partially by either the conformational changes to the backbone (VIII and IX) or the formation of an additional agostic interactions with Mn (X). Presence of the bulky groups at the R^2^ position causes decoordination of nitrogen, provides the NHC to act as a base, and leads to carbene–carbonyl coupling. Carbene–carbonyl coupling was always precluded by decoordination of the nitrogen from the Mn center and dissociation of the Mn–C bond. Such (−Mn–N, + C–O) series of events leads to stabilization by as much as −14 kcal mol^–1^. The complete set of CNC structures including −Mn–C reactivity is further analyzed in Section S4 of the Supporting Information. Figure S15 and Table S1 summarize all observed −Mn–C interactions (with no trimming based on relative energies). The stabilization by agostic interactions was detected with the H atoms from (one or two) ^*t*^Bu groups at R^1^ positions or even the C–H moieties from the pincer backbone (II, III, and IV). For the complexes bearing less bulky (OH and OCH_3_) adducts, their attack on the CO ligands was not accompanied by the pincer decoordination (“+C–O”, “+C–O, and +Mn–H”) (V and VI).

The favorable NHC–carbonyl coupling has been observed for the most bulky CNC complexes featuring the combination of R^1^=^*t*^Bu and R^2^=cy or ^*t*^Bu and X = Br or O^*t*^Bu (VIII–X, Figure 7). Such chemistry has been reported previously in several experimental studies. For example, Ruiz et al. explored the production of N-metalated NHC generated by the deprotonation of 1-phenylimidazole (L) in a cationic fac-[Mn(L)(CO)_3_(bipy)]^+^ complex and described a similar reactivity for the transient formation of carbene.^55^ It was established that the deprotonated imidazole and an auxiliary carbonyl ligand engaged in the mechanism of converting carbene into the more stable imidazolyl tautomer, which was responsible for the production of the acyl intermediate. It has also been shown that lithiated azoles can be added nucleophilically to [M(CO)_6_] (M = Cr, Mo, and W) to yield acyl intermediates that can then be alkylated to generate azolyl alkoxycarbene complexes.^56^ Huertos et al. reported on the intramolecular nucleophilic attack of deprotonated imidazoles to coordinated bipyridine and imidazole ligands.^57^ The new pathways for nucleophilic additions observed through high-throughput screening are of interest due to the high utility of catalytic transformations incorporating CO into organic substrates to create higher value products. Exemplary cases of such interactions include the hydroaminomethylation of simple vinylic arenes to produce a variety of useful pharmaceuticals in a one-pot reaction and the phosgene-free carbonylation of amino and phenolic compounds. The extent M–NHC catalysts will promote reactions that use CO as a C1-carbon source has been extensively discussed in the literature.^58,59^

### 4.3 Migrations of the CF_3_ Moieties

**Figure 8:**
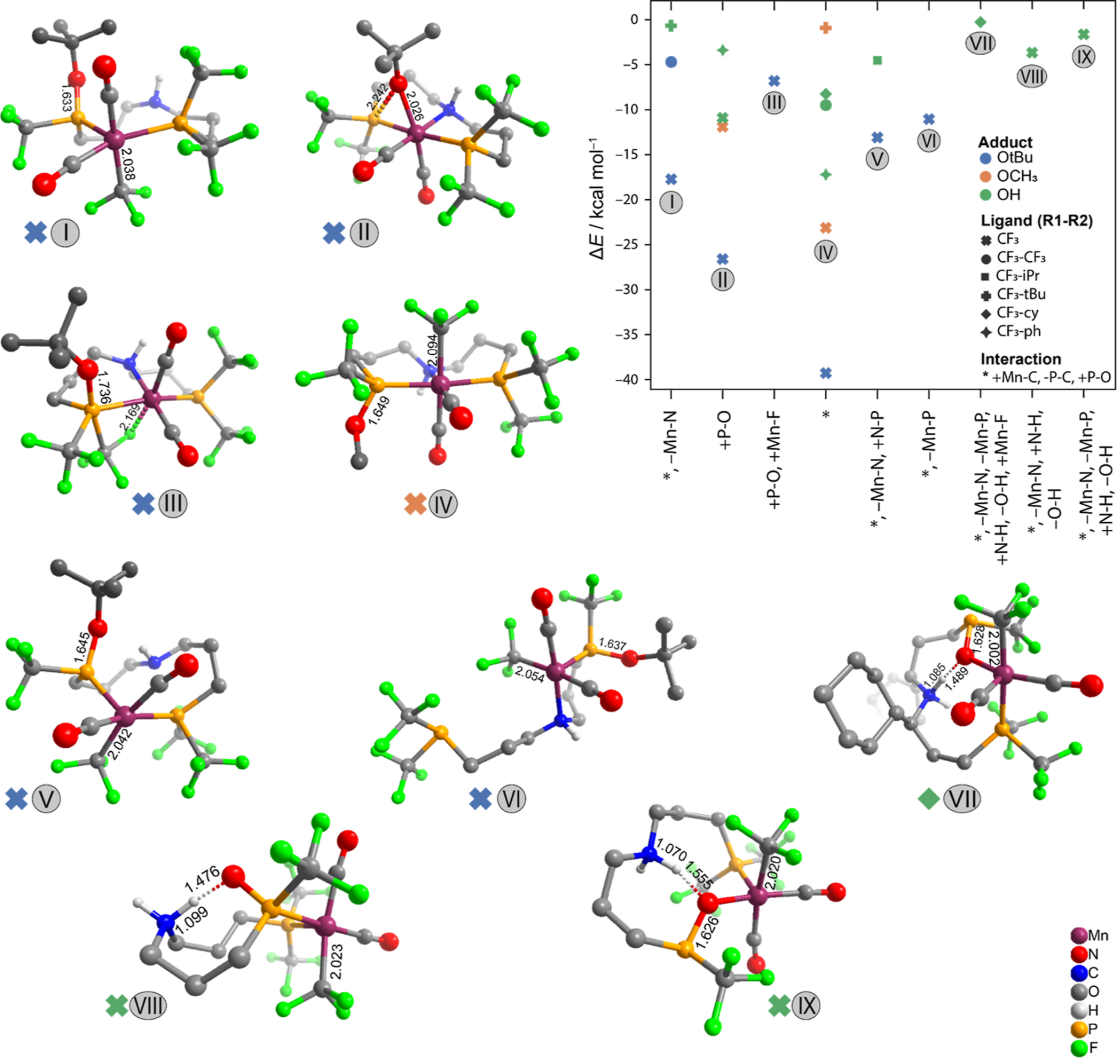
Formation of manganese carbonyl trifluoromethyl complexes for catalysts with a PNP backbone. The optimized structures of the most stable species from each class of interactions are presented (hydrogen atoms are omitted for clarity). Schematic representations of the most stable structures are presented in Figure S10 of the Supporting Information.

Another new reactivity was identified for the complexes featuring CF_3_-functionalized ligand scaffolds. The spontaneous migration of the originally P-bound CF_3_ moiety to the Mn center with concomitant exchange of the original alkoxide species has been identified. The most stable respective structures are summarized in Figure 8. Energetics of the observed structures are plotted on the *Y*-axis against the set of reactivities observed for catalyst structures with the PNP backbone shown on the *X*-axis. Symbols representing modifications at R^1^ and R^2^ positions as well as color coding for adducts present on the Mn center are shown in the legend. The set of reactivities summarized on the *X*-axis is briefly discussed below.

“*”: Summarizes a series of three events leading to the migratory insertion of the CF_3_ group to the Mn center including (I) the cleavage of the P-CCF_3_ bond (−P–C), (II) formation of the new P–O bond between P and the alkoxide (OH or O^*t*^Bu) adduct on the Mn center [+P–O(OH or O^*t*^Bu)], and (III) formation of the new Mn–CCF_3_ bond (+Mn–C).

Other reactivities described in Figure 8 along with the migratory insertion of CF_3_ include:

(*, −Mn–N) Structure I: For structures with OH and O^*t*^Bu adducts, decoordination of the nitrogen in the backbone has been observed along with the CF_3_ migration and leads to structures stabilized by ca.18 kcal mol^–1^.

**+P–O**: Structure II: Irrespective of the type of the adduct on the Mn center, it has been observed that the oxygen atom on the adduct can form a bridge with the adjacent phosphorus and can be an initial step for migration of CF_3_ groups.

**+P–O** and **+Mn–F**: Structure III: Migration of the adduct to the phosphorus and undercoordination of the Mn center are compensated by coordination with the F atom (III).

*, −Mn–P: Structure VI: Migration of the CF_3_ moiety to the Mn center has been observed to be accompanied by the dissociation of the Mn–P bond when there are no ligands present at R^2^ position (VI).

The structures formed via the migratory insertion are more stable than their reference structures within the [−39.2, −23.1] kcal mol^–1^ range of energies when no substitutions are present at R^2^ and within [−17.1 kcal mol^–1^, −0.8 kcal mol^–1^] when ph, CF_3_, cy, and ^*t*^Bu groups are present (Figure 8 and Table S6). Migratory insertion of CF_3_ onto the Mn center can be accompanied by cleavage of the Mn–N bond for the O^*t*^Bu and OH adducts. The adduct can be bridged between the Mn and P centers (initial step for migration of CF_3_ groups, Structure II, Figure 8). Migration of the adduct to the phosphorus is additionally stabilized by a short contact between Mn and the fluoride ion (III). The migration of CF_3_ onto the Mn center with concomitant exchange of the original alkoxide species was observed for OH and OCH_3_ adducts. This chemistry that we have identified purely from the expert-bias-free high-throughput computational reactivity analysis is in line with the previous studies of chemical systems with metal perfluoroalkyl bonds (M–RF) and the respective catalytic applications. Indeed, the more conventional organometallic compounds and especially metal alkyls (M–R) are immensely important players in catalysis.^60^ Catalysis utilizing metal fluoroalkyl complexes, however, is less common due to the inherent stability of M–RF.^61^ Such compounds, on the other hand, receive increasing attention for their utility in the field of fluoro-organic synthesis.^62,63^ For example, [Cu]–RF compounds are utilized as the stoichiometric reagents for perfluoroalkyl transfer to organic substrates.^64–67^ Recently, there is much attention to transition-metal-catalyzed (with such metals as Cu, Ni, and Pd) C–RF (where RF is usually CF_3_) bond-forming reactions,^68,69^ providing a route to various valuable fluorinated pharmaceuticals and agrochemicals.^62,63,70^ Daniels et al. discussed the synthesis, characterization, and reactivity of several bi- and tridentate, N-ligated manganese carbonyl trifluoromethyl complexes.^61^ All of these complexes feature elongated Mn–CF_3_ bonds, suggesting the lability of the moiety, which could potentially be exploited for the transfer or insertion of the CF_3_ group into organic substrates. Poli and co-workers investigated in detail the thermal decarbonylation of the acyl compounds [Mn(CO)_5_(CORF)] (RF = CF_3_, CHF_2_, CH_2_CF_3_, and CF_2_CH_3_) and the formation of [Mn(CO)_5_(RF)] species containing M-alkyl moieties.^71^ The Mn–RF moiety is highly labile and can undergo homolytic dissociation upon moderate heating or when subjected to photochemical (UV or visible light). For example, such activation procedures allow [Mn(CO)_5_(CF_3_)] compound with the strongest Mn–RF bond to initiate the radical polymerization in the synthesis of poly(vinylidene fluoride).^71^ The migration of the CF_3_ moiety to the Mn center identified by the current automated algorithm is thus in line with the previous experimental investigations on the related chemistries and suggests new avenues to expand this field by utilizing CF_3_-modified ligand scaffolds and secondary transformations of the respective Mn catalysts.

## 5 Conclusions

A method is introduced for the automated exploration of the reactivities of extended databases of transition-metal catalysts. The proposed workflow is designed to tackle the key challenges for bias-free mechanistic explorations on large databases of catalysts, namely: (I) the automated exploration of the chemical space around each catalyst given specific structural features and (II) the automated analysis of results from such chemical data sets and provision of design rules for catalyst with improved performances or new reactivity. To address these challenges, we have extended the application of our previously developed ReNeGate method for bias-free chemical space exploration to databases of synthetic organometallic catalysts. We implemented an analysis procedure to identify the classes of reactivity patterns within specific catalyst groups in the large organometallic data sets. Our procedure applied to an extended series of representative Mn(I) pincer complexes revealed new correlations between the structural and reactive features, pointing to new channels for catalyst transformation under the reaction conditions. Specifically, we have identified different hemilability behaviors for catalysts with CNC, PNN, PNP, and SNS backbones with a data-driven approach. Understanding hemilability is important because it affects the energy changes associated with preactivation and regeneration steps in the catalytic process and, in turn, influences the coordination sphere and geometry of the complex. In addition, two new classes of reactivities, namely, nucleophilic attack on carbonyl ligands and migration of CF_3_ moieties have been identified through high-throughput virtual screening on the databases. Such a bias-free high-throughput virtual screening on the systematically designed structures opens new opportunities for the design of high-performance catalysts as well as an accelerated method for exploring new reactivity patterns.

## Data Availability

Source code and dataset for HiREX are freely available at: https://github.com/ahashemiche/HiREX. HiREX uses ReNeGaTe and ChemSpaX libraries that are freely available at: https://github.com/ahashemiche/ReNeGate and https://github.com/EPiCs-group/chemspax.

## Supporting Information Available

S1 Relevant molecular graph theory terminology; S2 Sample reaction network analysis with ReNeGate and comparison against reference structure using HiREX; S3 Correlation analysis of the structural features and reactivity for explored structures: Energetic distribution of explored structures, Correlation analysis of the structural features and reactivity for structures in [−40, 25] kcal mol^–1^ range; Role of adducts in observed reactivities within [0, −40] kcal mol^–1^; Schematic representations of the optimized structures shown in Figures 7 and 8; Exploration results leading to decoordination of Mn–N ligands for catalysts with different backbones; Exploration results for attack by NHC moieties of the CNC pincer on the carbonyl ligands (Mn–C interactions); S4 Supplementary tables: Tabular data for DFT-optimized structures for explored species (PDF)
XYZ structures for reference structures, explored structures from Table S7 and excel file for supplementary tables (including structure IDs, structural features, energetics, and reactivities) (ZIP)
