# Supplementary material for: HiREX: High-Throughput Reactivity Exploration for Extended Databases of Transition-Metal Catalysts
Source: J Chem Inf Model. 2023 Sep 22;63(19):6081–94. doi: 10.1021/acs.jcim.3c00660 (PMC10565810; doi:10.1021/acs.jcim.3c00660)
Supplement: Supplementary file 1 — ci3c00660_si_001.pdf [file ci3c00660_si_001.pdf]

Supporting Information: HiREX: High-Throughput Reactivity Exploration for Extended Databases of Transition Metal Catalysts

Ali Hashemi,[*,†] Sana Bougueroua,[‡] Marie-Pierre Gaigeot,[‡] and Evgeny Pidko[*,†]

†*Inorganic Systems Engineering, Department of Chemical Engineering, Faculty of Applied Sciences, Delft University of Technology, Van der Maasweg 9, 2629 HZ, Delft, The Netherlands*

‡*Laboratoire Analyse et Modelisation pour la Biologie et l'Environnement (LAMBE) UMR8587, Paris-Saclay, Univ Evry, CY Cergy Paris Universite, CNRS, LAMBE UMR8587, Evry-Courcouronnes 91025, France*

E-mail: ahashemiche@gmail.com; E.A.Pidko@tudelft.nl

S1 Relevant Molecular Graph Theory Terminology

Definitions given in section S1 are fundamental terms needed to understand the chemical fingerprinting procedure and are reproduced from our previous article on the ReNeGaTe methodology with permission from reference S1. Copyright © 2022 American Chemical Society.

- **Graphs:** A graph **G** is defined as $\mathbf{G} = (\mathbf{V}, \mathbf{E})$, where **V** represents the set of **vertices** (also called nodes or points) and **E** represents the set of all **edges** (also called links) in the graph. We distinguish many types of graphs (Figure S1):

    1. **Directed graph**: a graph $\mathbf{G} = (\mathbf{V}, \mathbf{A})$, where all the edges are **directed** from one vertex to another. The edges are in general called **arcs**.
    2. **Undirected graph**: a graph $\mathbf{G} = (\mathbf{V}, \mathbf{E})$, where all the edges are **bidirectional.**
    3. **Mixed graph**: a graph $\mathbf{G} = (\mathbf{V}, \mathbf{E}, \mathbf{A})$ consisting of a set of **undirected** edges **E**, and a set of **directed** edges (arcs) **A**.

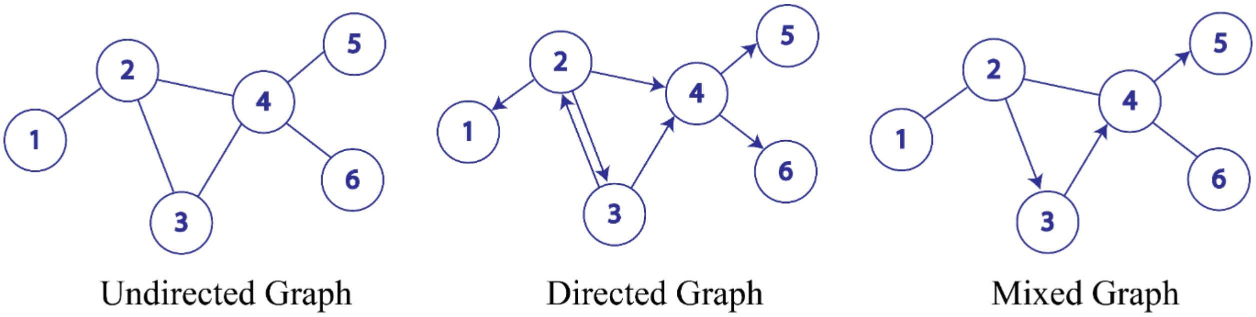

Figure S1: Graph Types based on directionality of edges

Recent implementations of graph theory in chemistry and statistical analysis of MD trajectories have shared a similar degree of granularity in representing molecular systems using simple graphs, where a vertex represents an atom or a molecule and edges represent the interactions/bonds (covalent bond, hydrogen bond...). Following are some definitions of a molecular conformation and their relationship to graph theory:

- **Molecular Graph**: One crucial step of the algorithm is to set up a model that defines a molecular conformation with the right level of granularity. In our case, we have chosen to define the configuration in terms of covalent bonds and organometallic and ionic interactions formed between the atoms. The definitions are based on Euclidean distances as described below:

- - **A bond**: is formed between a pair of atoms [a, b] with respective Cartesian coordinates (x_a, y_a, z_a) and (x_b, y_b, z_b), if the Euclidean distance

$$dist = \sqrt{(x_a - x_b)^2 + (y_a - y_b)^2 + (z_a - z_b)^2} \tag{S1}$$

is less than a cut-off distance D_r. For covalent bonds, the algorithm defines the D_r distance by the sum of covalent radii of atoms a and b with an additional margin of 2% of this sum. For the organometallic and ionic interactions, the expert user is given the freedom to set case specific Dr distances, for example, the distance between manganese and oxygen atoms (2.44 Angstrom), etc. This choice was made because the developers assume that the covalent bonds are stronger than the other types of interactions between atoms.

By defining the different bonds, graphs corresponding to different conformations are constructed. By definition, a conformation is translated into a mixed graph

$$G = (V, E_C, A_H, E_I, E_O) \tag{S2}$$

where:

  - **V**: is the set of all atoms present in the conformation. Each atom represents one vertex in the graph G.
  - $\mathbf{E}_C$: the set of covalent bonds. Each covalent bond represents an undirected edge in the graph G.

- $\mathbf{A}_H$: the set of hydrogen bonds. Each hydrogen bond represents a directed edge in the graph G.
- $\mathbf{E}_I$: the set of intermolecular/ionic interactions. Each intermolecular/ionic interaction represents an undirected edge in the graph G.
- $\mathbf{E}_O$: the set of organometallic interactions. Each organometallic interaction represents an undirected edge in the graph G.

The Cartesian atomic positions taken from the trajectory are used only for forming the mixed graphs. Once the graphs are obtained, the changes in the conformations are analyzed through the comparison of these graphs using isomorphism check.

- **Molecular Graph isomorphism**:

Molecular configurations are translated into unique graphs based on threshold values (Equation S1). Once conceived as graphs, isomorphism check is done to track possible changes along the trajectory. To reduce the cost of the isomorphism tests, optimizations were used as defining orbits around the atoms.[1] For the present work, the algorithm is developed for analyzing molecular dynamics trajectories in terms of arbitrary bonding types which represent the conformational change.

Two conformations are different if and only if they are not isomorphic. The isomorphism between two graphs is defined by a **bijection** between them: Two graphs G_a and G_b are **isomorphic** if and only if there exists a **bijection** $\theta_{a,b} : V_a \quad V_b$ such that :

- $\forall\ v\ \in V_a,\ \emptyset\,(v) =\ \emptyset(\theta_{a,b}\,(v)),\ \theta_{a,b}\,(v) \in V_b$
- $[v, u] \in E_{c_a} \Leftrightarrow\ [\theta_{a,b}\,(v)\,,\ \theta_{a,b}\,(u)] \in\ E_{c_b}$
- $(v, u) \in A_{H_a} \Leftrightarrow\ (\theta_{a,b}\,(v)\,,\ \theta_{a,b}\,(u)) \in\ A_{H_b}$
- $[v, u] \in E_{I_a}\ \Leftrightarrow\ [\theta_{a,b}\,(v)\,,\ \theta_{a,b}\,(u)] \in\ E_{Ib}$
- $[v, u] \in E_{O_a} \Leftrightarrow\ [\theta_{a,b}\,(v)\,,\ \theta_{a,b}\,(u)] \in\ E_{Ob}$

In our case, we apply an isomorphism test considering the atom chemical type as an attribute for different nodes. Consequently, each atom will be given a *color*. Two atoms can be exchanged if and only if they have the same color. Using this partitioning will not only allow a coherent comparison between graphs but also reduce the number of possible permutations to decide if the graphs are identical or not and thus the algorithm can perform faster. Figure S2. illustrates three example graphs where graphs (a) and (b) are isomorphic, while graph (c) is not isomorphic to a and b.

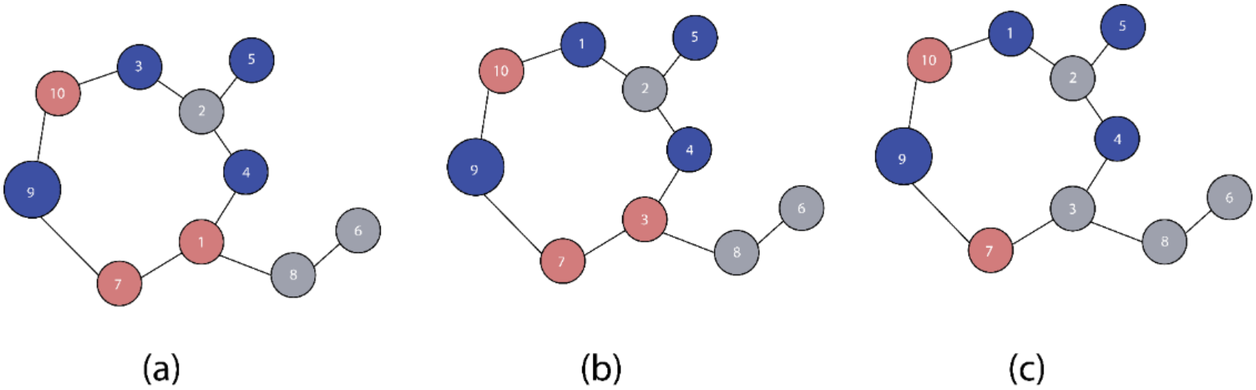

Figure S2: Using the isomorphism conditions, graphs a and b are considered isomorphic while graph c is not isomorphic to them

A molecular system is defined as a graph such that vertices represent atoms of the molecular system and edges represent the bonds formed between these atoms (covalent bond, hydrogen bonds, intermolecular electrostatic interactions, etc., depending on the system). Conformational dynamics of molecular system do occur (evidently depending on internal energies and energy barriers on the potential energy surface), with ”fastest motions” being hydrogen/ionic bond dynamics (forming/breaking) while larger amplitude motions can induce large structural modifications such as torsional movements. Chemical reactions are defined as the occurrences of formation/breakage (covalent) bonds, leading to numerous changes in the chemical entities over time. Bond dynamics (forming/breaking) represent, in the graph terminology, a change in the edge sets. The exploration of different configurations can be seen as an exploration of differ-

ent graph topologies, tracked using graph theory methods via checking for isomorphic graphs in the configuration ensemble.[2] An isomorphism between two graphs is a bijection between their vertex sets that preserves adjacency3, in other words, it is a function between the elements of two sets, where each element of one set is paired with exactly one element of the other set, and each element of the other set is paired with exactly one element of the first set.

Isomorphism checks are the key components of the *reaction event exploration* step and are used to identify unique conformations from the reactive trajectory. Based on the time evolution of the unique conformers in the reactive trajectory, a graph of transition (showing how the conformations are related one to another and the time sequence) for species present in the reference network is formed. The changes in conformations are followed over time evolution of trajectories for changes in bonding patterns of choice (among hydrogen bond(s), proton transfer(s), coordination number(s), covalent bond(s) and organometallic interaction(s)).

S2 Sample reaction network analysis with ReNeGate and comparison against reference structure using HiREX

In order to better understand the underlying algorithms to find unique structures and generation of reaction networks, we refer the interested reader to our previous publication on the ReNeGaTe workflow.[3] Here we present a sample reaction network analysis and followup comparison of the observed unique structures against reference (starting) structure. As observed in Figure S3.a, a reaction network is generated where nodes represent unique chemical species observed in the trajectory. Edges represent chemical transformations observed between different nodes. Edges are labeled with the frequency of the observed transformations.

After trimming the reaction network based on the assumed threshold values for nodes and edges, the observed structures are compared against the respective reference structure and changes in bonding pattern in comparison with the reference are recorded for further analysis in the high-throughput reactivity exploration database. (Figure S3.b)

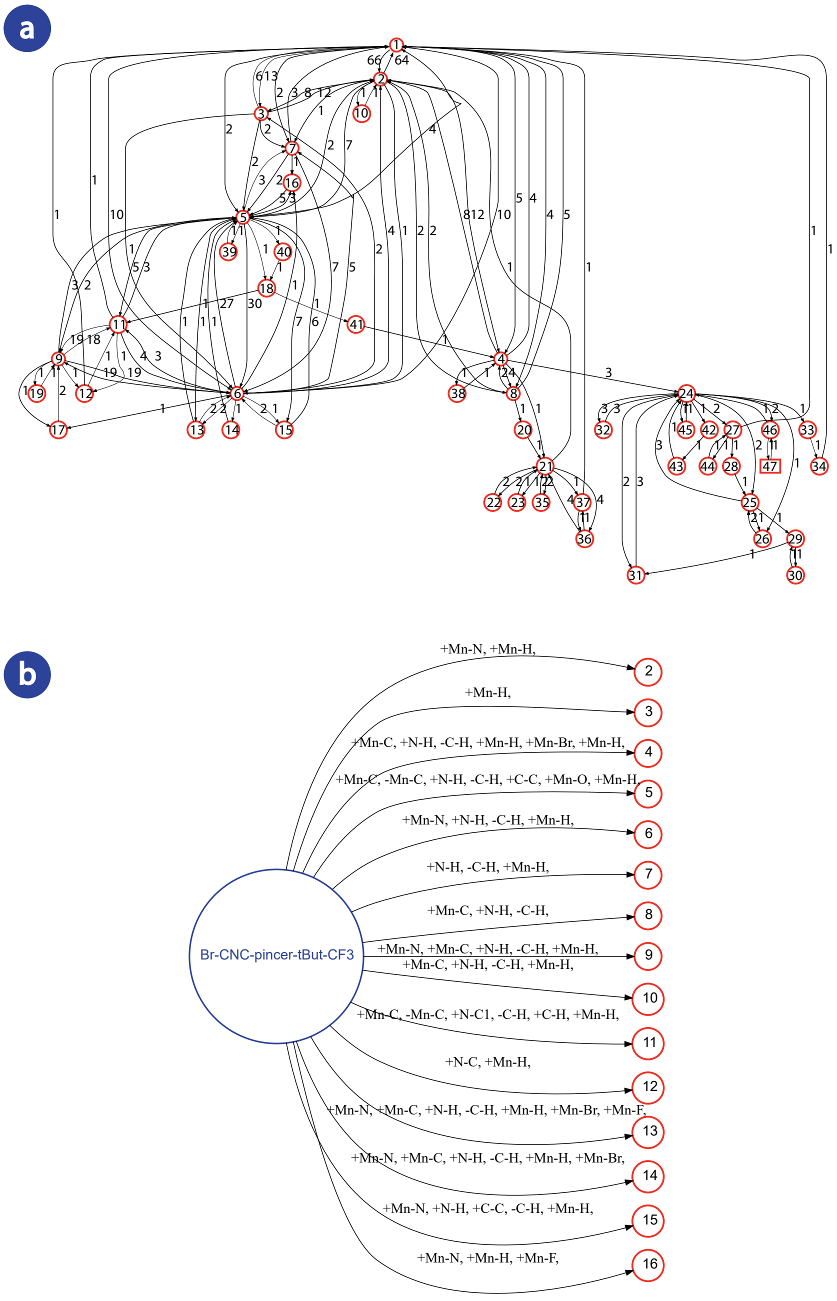

Figure S3: a)Sample analysis of a reactive trajectory using ReNeGaTe b) further comparison of observed reactivities against reference structure

S3 Correlation analysis of the structural features and reactivity for explored structures

Figure S4 and table S1 explain the energetic distribution of explored structures. For catalysts with CNC, PNN, PNP and SNS backbones, the fraction of structures found within [-40,25] kcal.mol^{-1} range with respect to all explored structures with the specific backbone are respectively [48%, 91%, 48%, 70%]. This shows that our exploration methodology has been successful in exploring different regions of the potential energy surface.

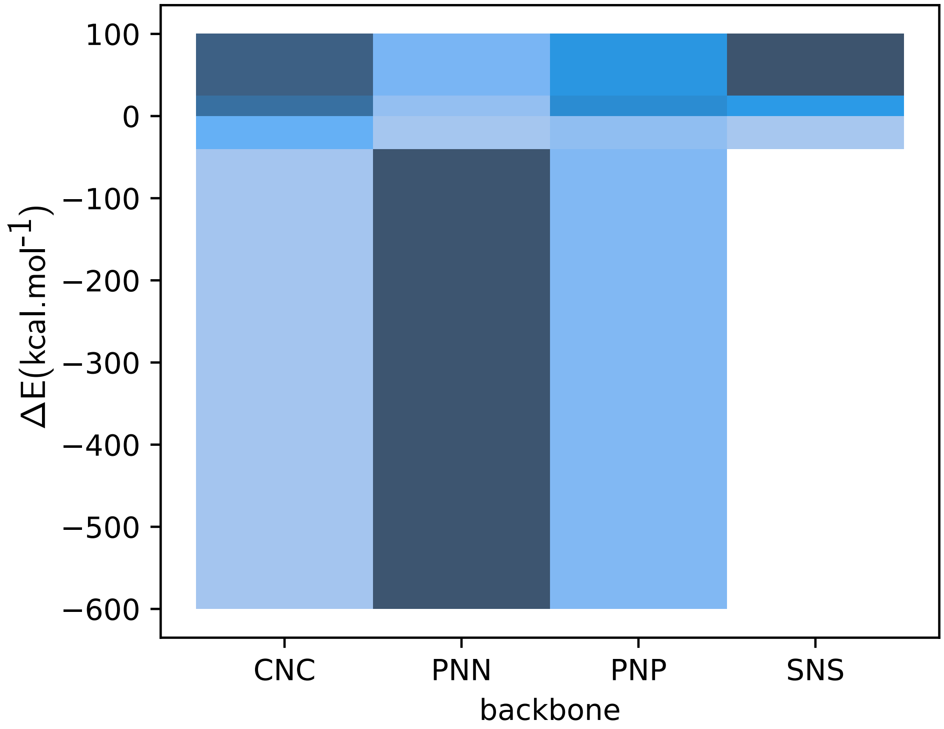

Figure S4: Histogram plots for the energetic distribution of explored structures in the [-600, 100] kcal.mol^{-1} range based on the backbone structures

Table S1: Energetic distribution of explored structures for catalysts with CNC, PNN, PNP and SNS backbones: Number of explored structures and respective percentages of all explored structures are reported in [-600,-40] kcal.mol^{-1} [-40, 0] kcal.mol^{-1} [0,25] kcal.mol^{-1} [25, 150] kcal.mol^{-1}

	**[-600,-40]**	**[-40, 0]**	**[0,25]**	**[25, 150]**	**Total**
**CNC**	8	56	189	222	475
**PNN**	245	7	23	44	319
**PNP**	39	26	132	112	309
**SNS**	0	5	104	250	359
**Total**	292(20%)	94(7%)	448(30%)	628(43%)	1462

The trends observed for R^1- (backbone, R^2, CN, ΔE, X) combinations in the [-40, 0] kcal.mol^{-1} range are also generally observed in the [-40,25] kcal.mol^{-1} range. Additional observations limited to only [0,25] kcal.mol^{-1} for R^1-backbone combinations include (H, Ph)-PNN , (H,cy,Ph)-PNP, (^{t}Bu,cy,Ph,^{i}Pr)-SNS. Presence of bulky (Ph,cy,^{t}Bu and ^{i}Pr) moieties at R^1 can give rise to unstable alternative structures. R^1-R^2: (ph,^{i}Pr)(H,ph), cy-ph, H-(^{i}Pr,^{t}Bu) combinations are also limited to structures within [0,25] kcal.mol^{-1}. 7-coordinated Mn is observed also only in [0,25] kcal.mol^{-1} when cy is present at R^1. For different R^1-X combinations Br-(ph,^{i}Pr), O^{t}Bu-(H,^{i}Pr), OCH_3-ph combinations only lead to structures with higher energies than the respective reference.

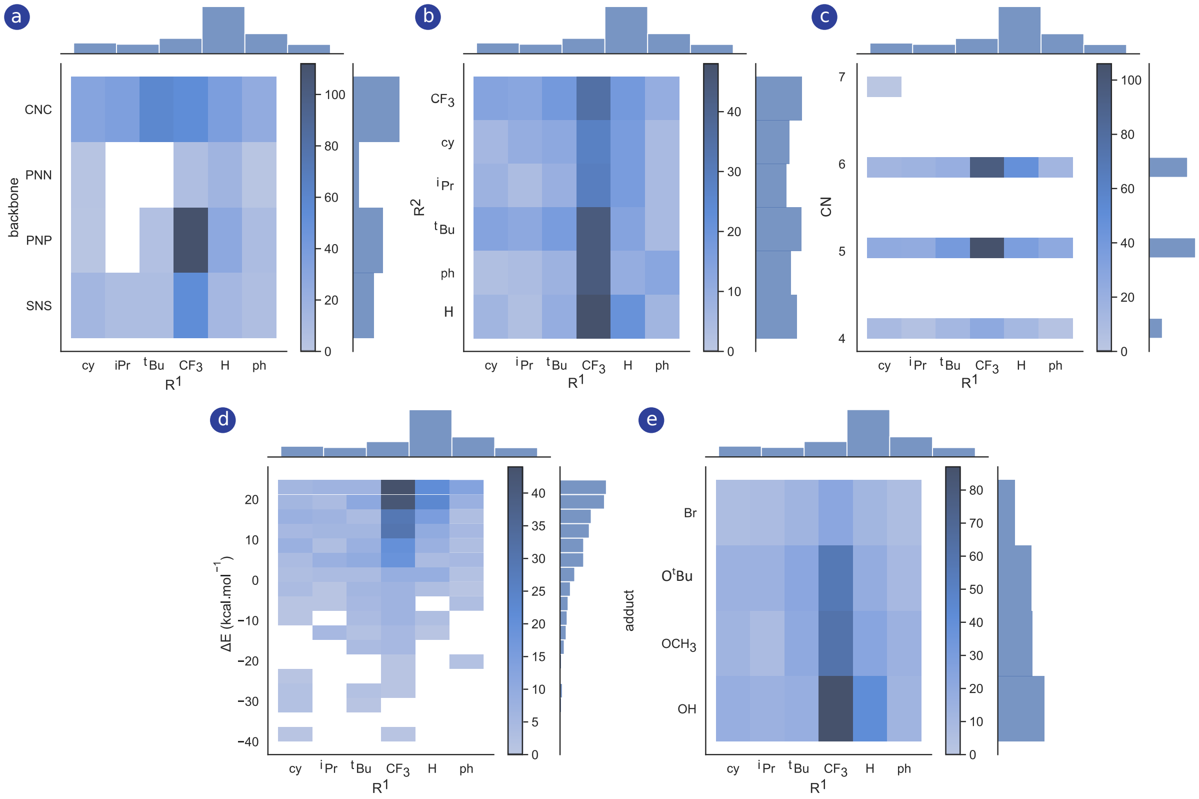

Figure S5: Histogram plots for the frequency of appearance of alternative stable species in the [-40,25] kcal.mol^{-1} range based on modified structures for CNC, PNP, PNN and SNS catalysts as a function of feature combination (a) R^1 – backbone, (b) R^1 – R^2, (c) R^1 – CN (coordination number of the metal center) (d) R^1 – ΔE (relative energy) and (e) R^1-X.

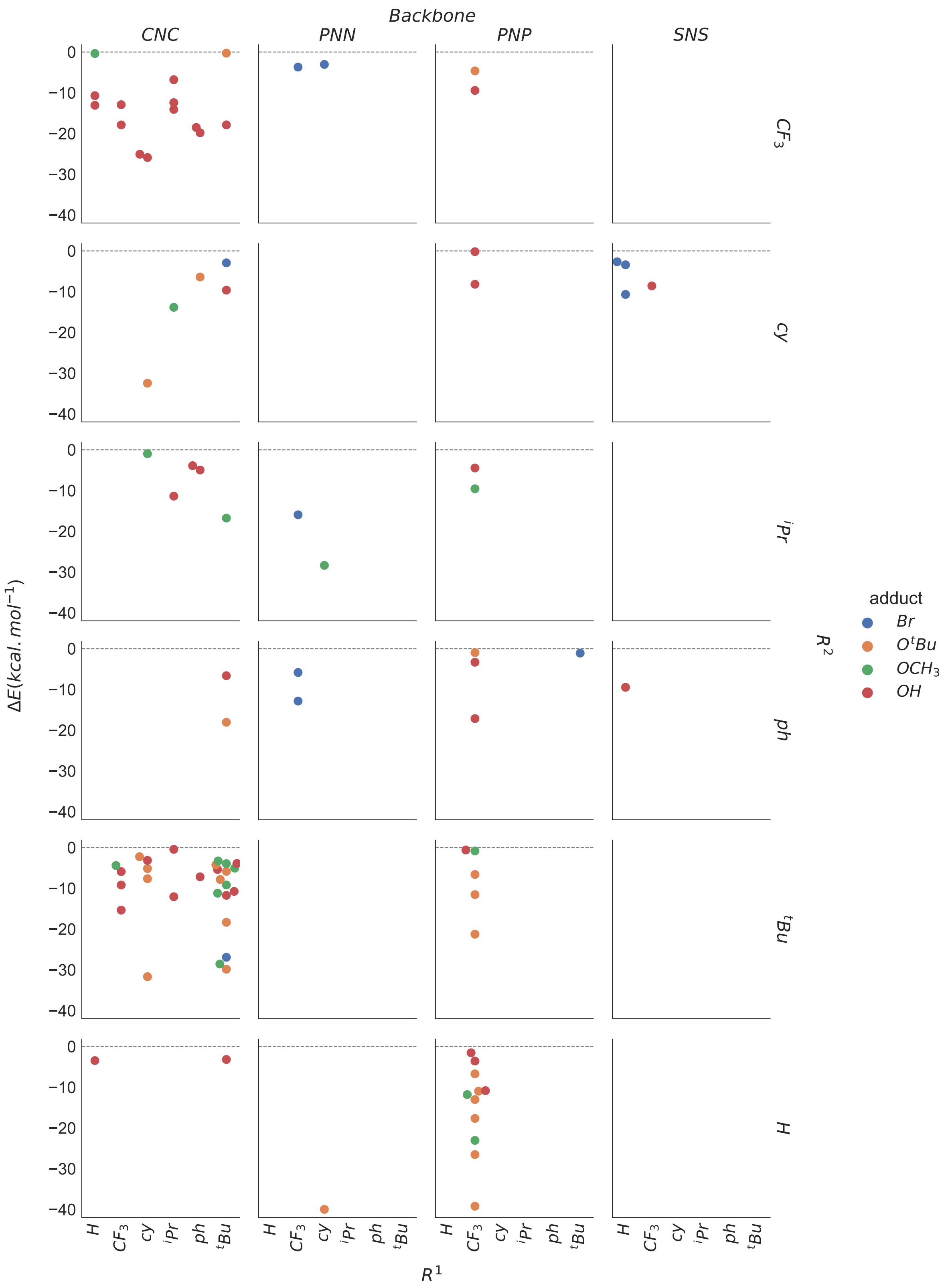

Figure S6: Exploration results for deactivated species. Species with lower energies compared to respective reference structures are categorized based on the R^1, backbones and adducts. Nodes are colored based on R^2 ligands.

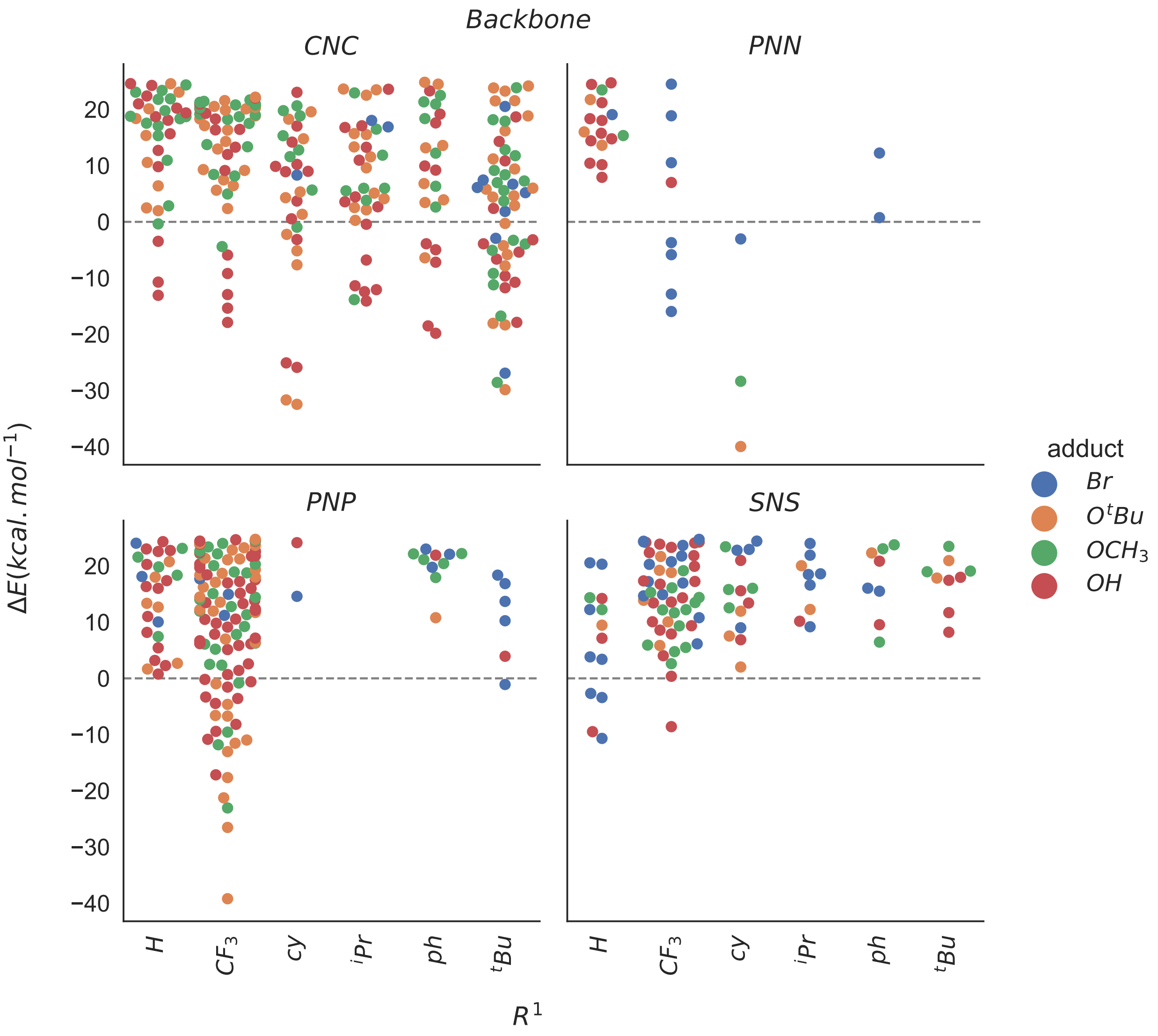

Figure S7: Explored results for species less than 25 kcal.mol^{-1} different in energy from the respective reference structures. Species are categorized based on the ligand substituent on the R^1 position on the backbone of the catalyst for different backbones and adducts on the Mn center.

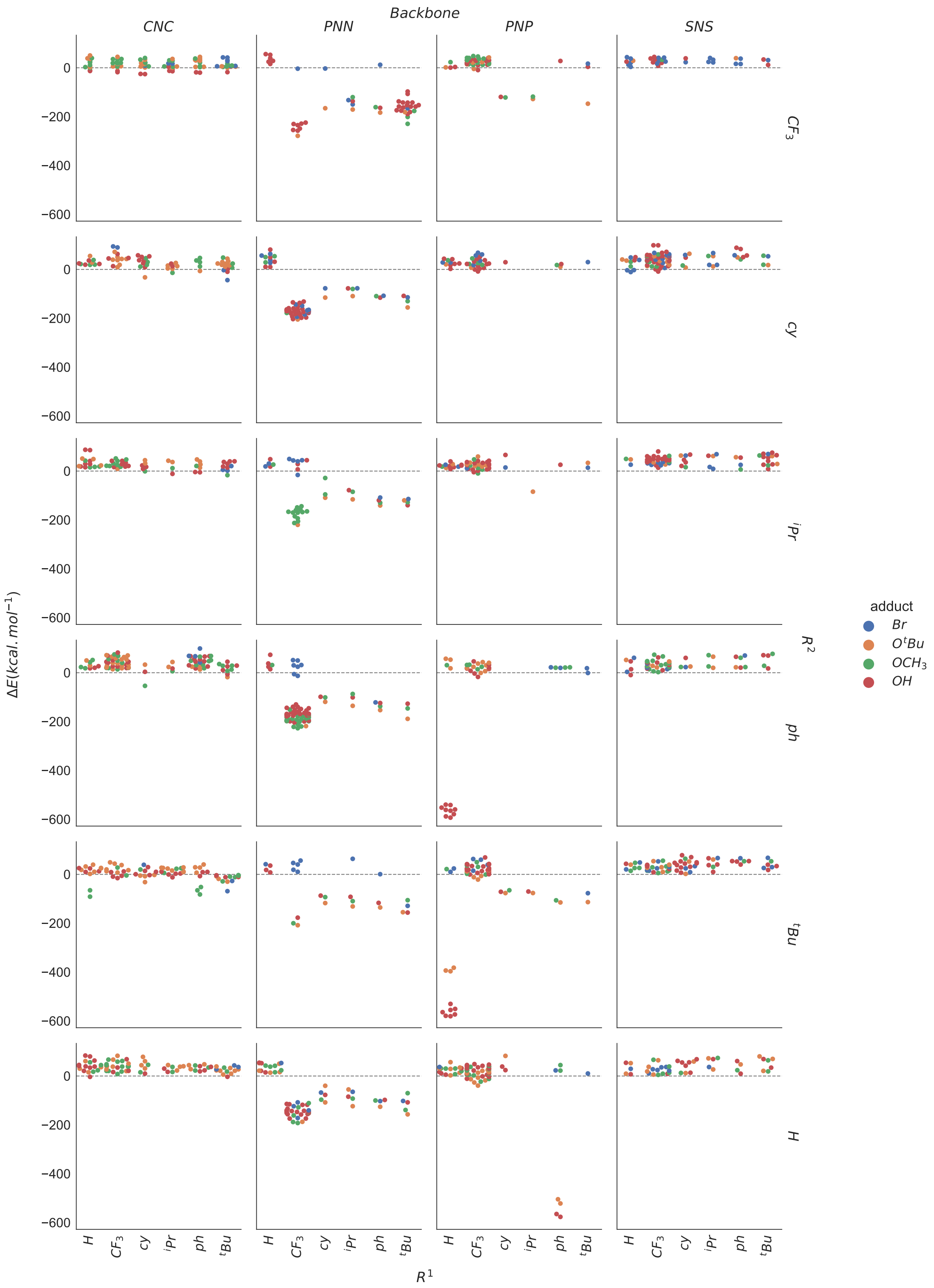

Figure S8: Exploration results for all explored species categorized based on R^1 and R^2 ligands, adduct and backbone. Datapoints colored based on the adduct present on Mn.

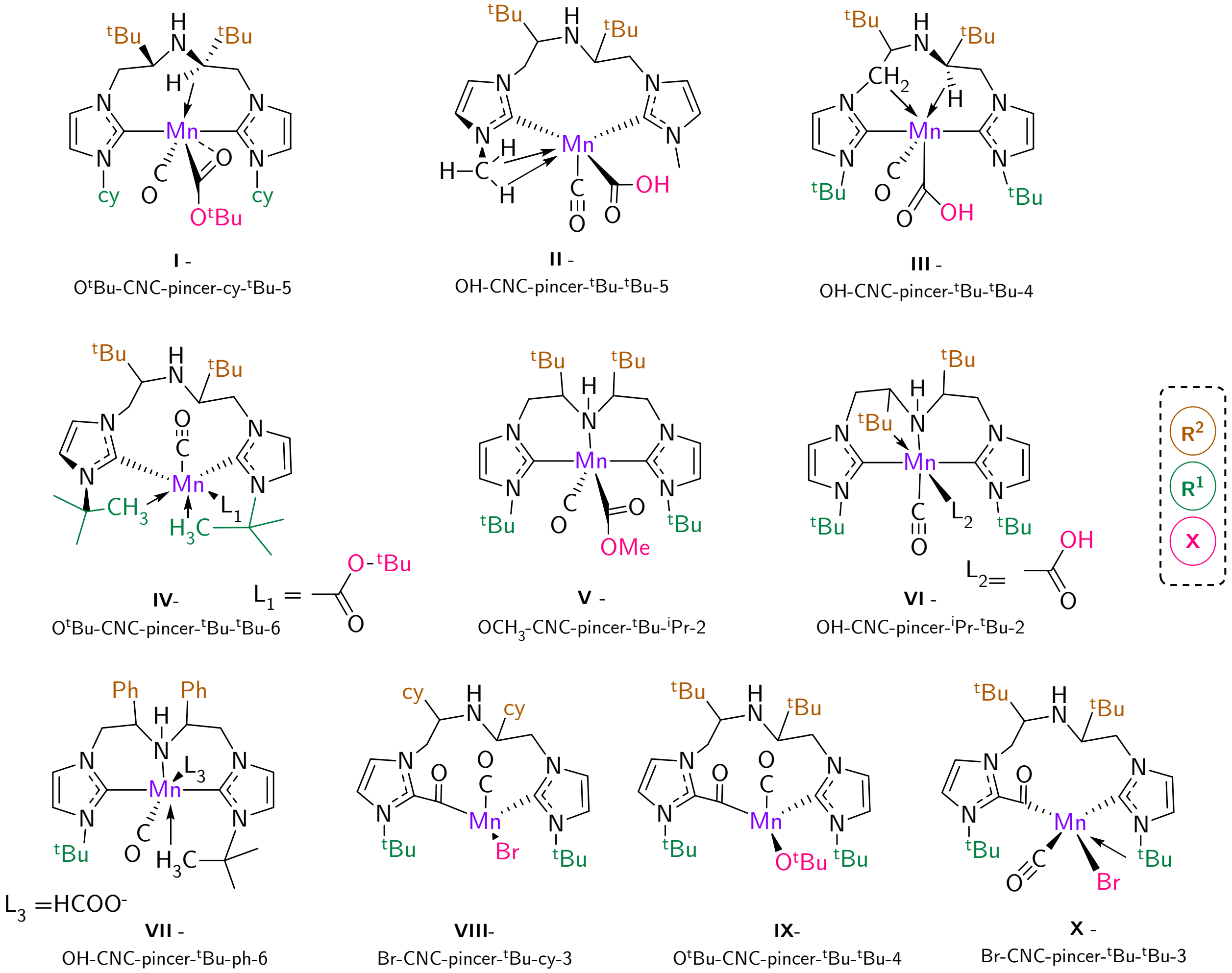

Figure S9: Schematic representations of the optimized structures shown in Figure 7 of the manuscript: Ligand substitutions at R^1 and R^2 positions as well as the adduct are colored according to the legend for clarity

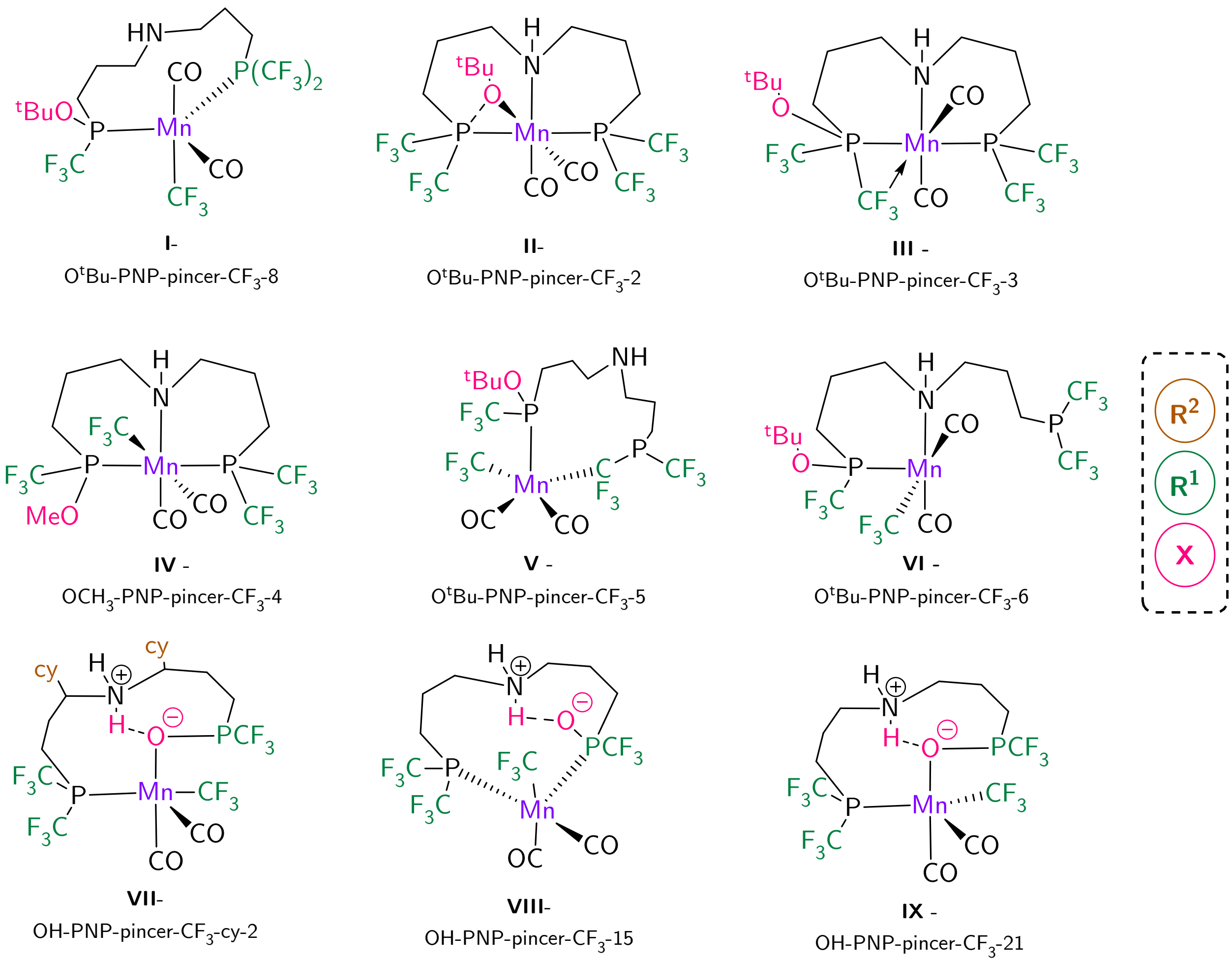

Figure S10: Schematic representations of the optimized structures shown in Figure 8 of the manuscript: Ligand substitutions at R^1 and R^2 positions as well as the adduct are colored according to the legend for clarity

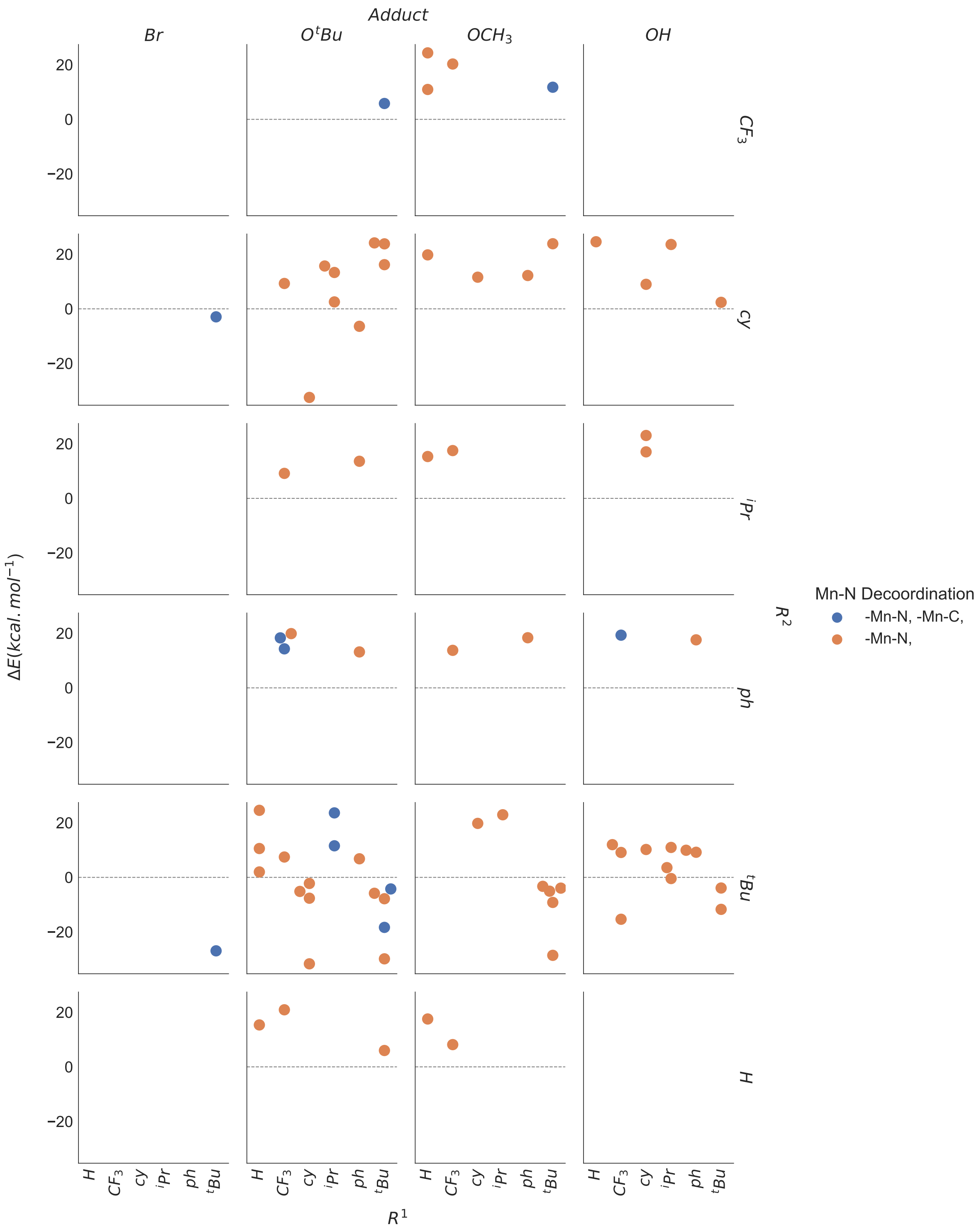

Figure S11: Mn-N decoordination for catalysts with CNC backbone: Presence of bulky ^{t}Bu and cy functional groups at R^2 position leads to the decoordination of the Mn-N bond. When CF_3 or no functional groups are present at R^2 position along with H or CF_3 groups at R^1 position –Mn-N is observed. Mn decoordination is not observed when Br is coordinated to the Mn center.

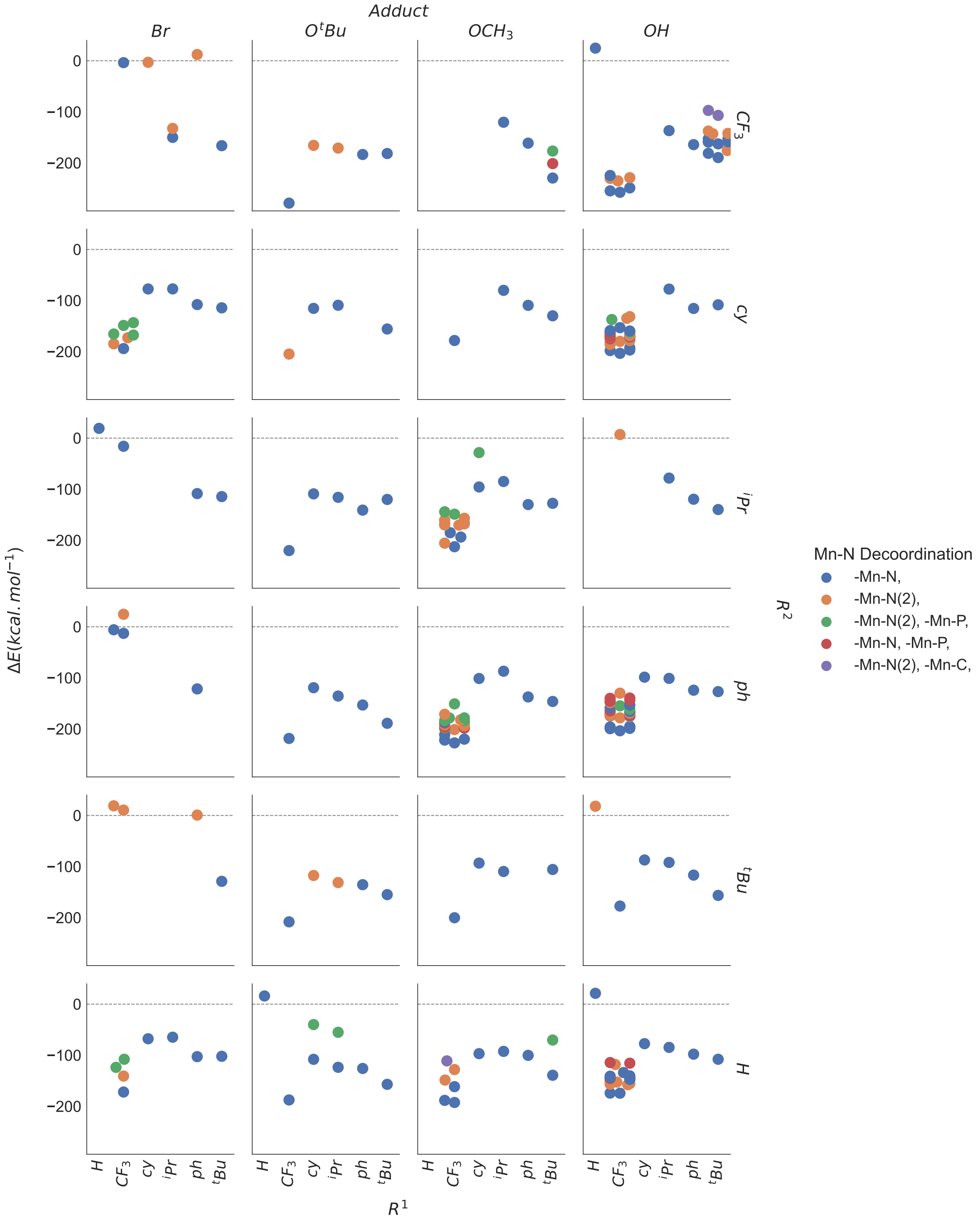

Figure S12: Mn-N decoordination for catalysts with PNN backbone: presence of CF_3 groups at either of R^1 or R^2 position has a direct impact on the decoordination of the Mn-N bond. Mn-N bond very easily decoordinates with all R^1-R^2-Adduct combinations. Decoordination of both Mn-N bonds, -Mn-N, -Mn-P or complete decoordination of the ligands are specific to the presence of CF_3 groups at R^1 position.

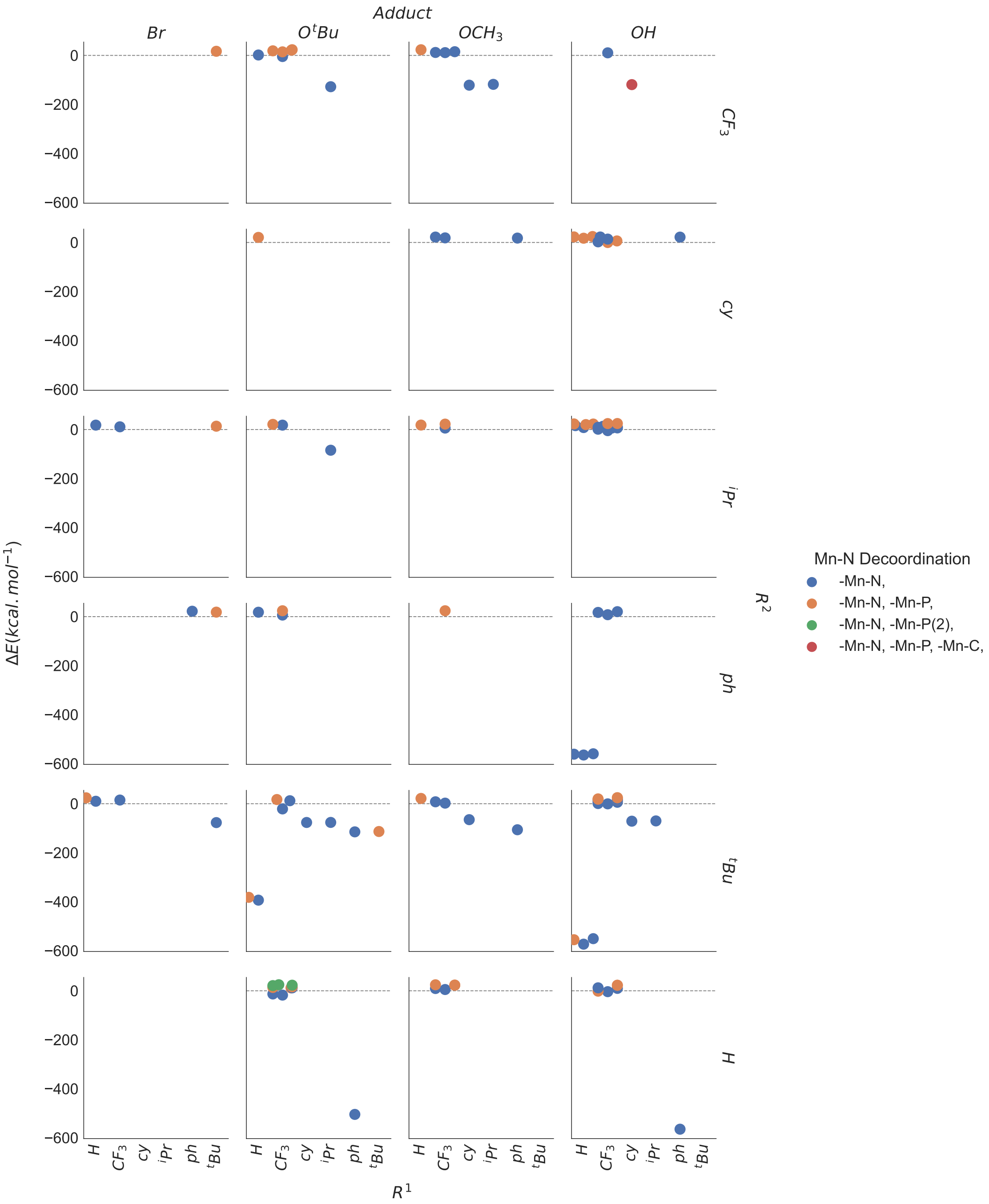

Figure S13: Mn-N decoordination for catalysts with PNP backbone: decoordination behaviors observed include decoordination of the -Mn-N bond along with either or both of Mn-P ligands donors. While decoordination of the Mn-N bond is observed for all R^1 -R^2 combinations, decoordination of one Mn-P is observed when H or CF_3 groups are present at R^1 position. Complete dissociation of all backbone ligands is observed when CF_3 groups are present at R^1 and no R^2 (R^2 = H) modifications are made.

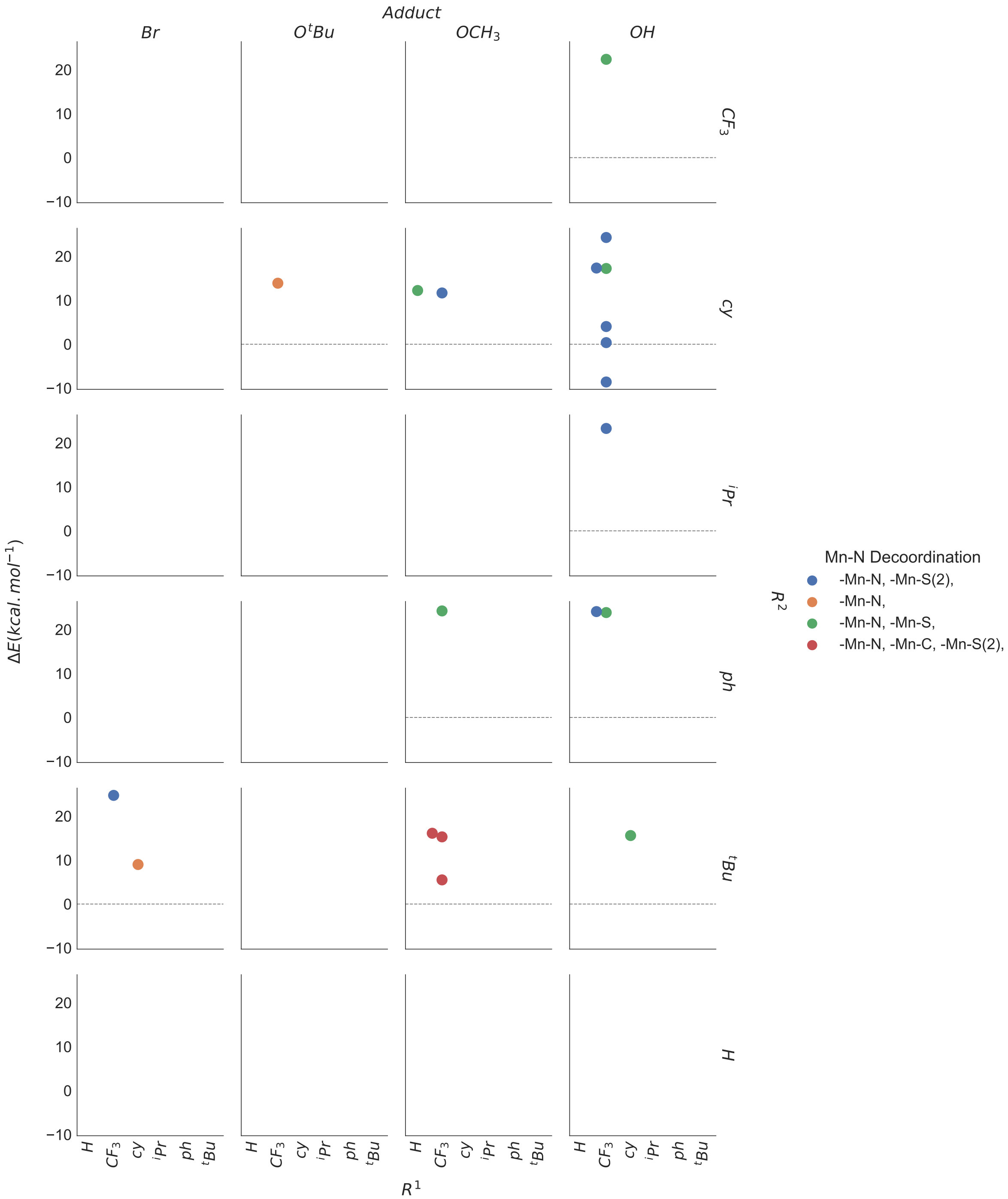

Figure S14: Mn-N decoordination for catalyst with SNS backbone: For catalysts with SNS scaffolds, decoordination behaviors observed include decoordination of the -Mn-N bond along with either or both of Mn-S ligands donors. Presence of CF_3 groups at R^1 position has a clear impact on the decoordination of both Mn-N and Mn-S donor ligands. Decoordination of the central Mn-N with R^1: CF_3 and R^2:cy.

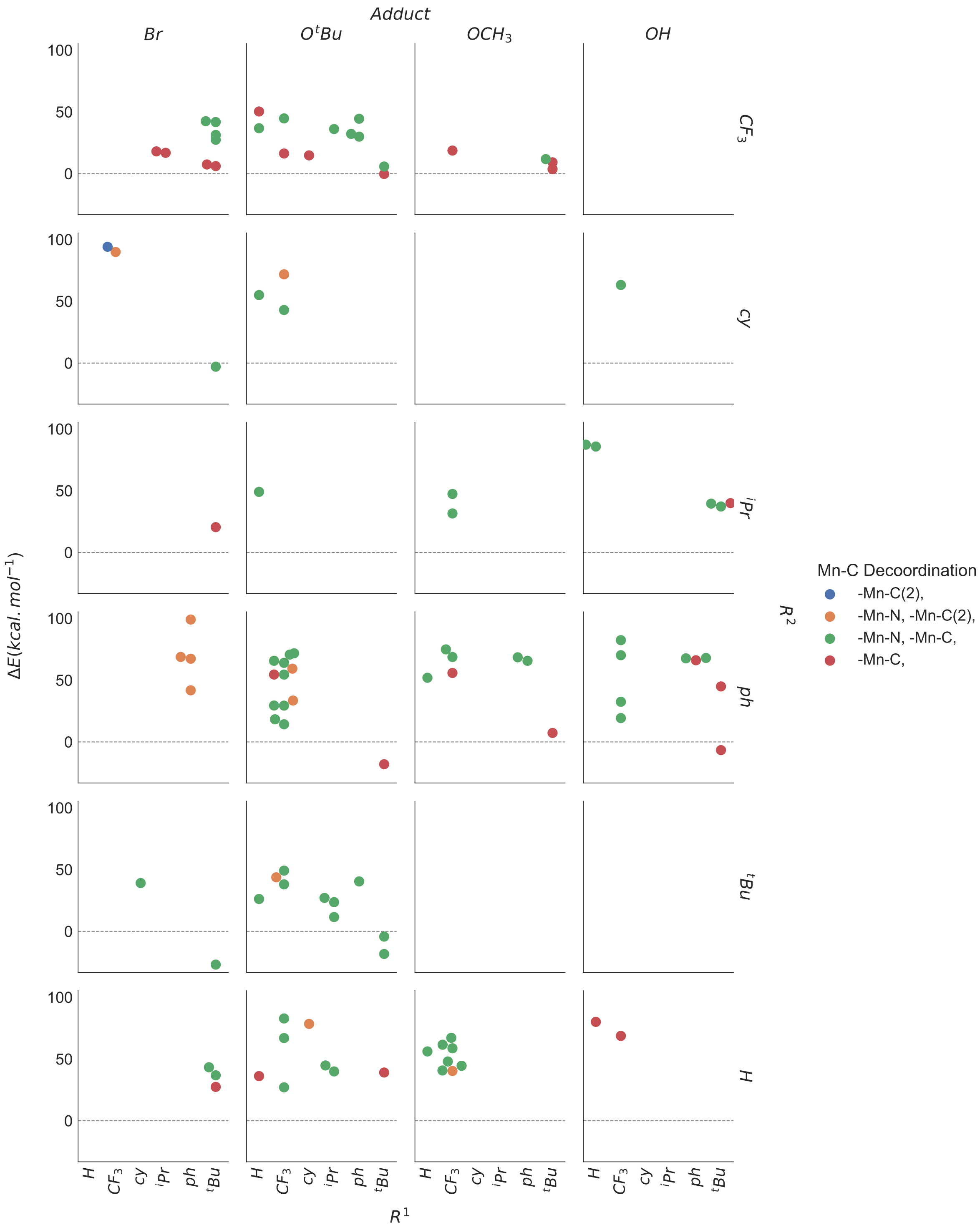

Figure S15: Mn-C decoordination for catalyst with CNC backbone: Presence of bulky ^{t}Bu groups at R^1 positions leads to the decoordination of the Mn-C along with decoordination of the Mn-N bond. Presence of electron withdrawing CF_3 groups at R^2 position also causes the decoordination of the Mn-C bond.

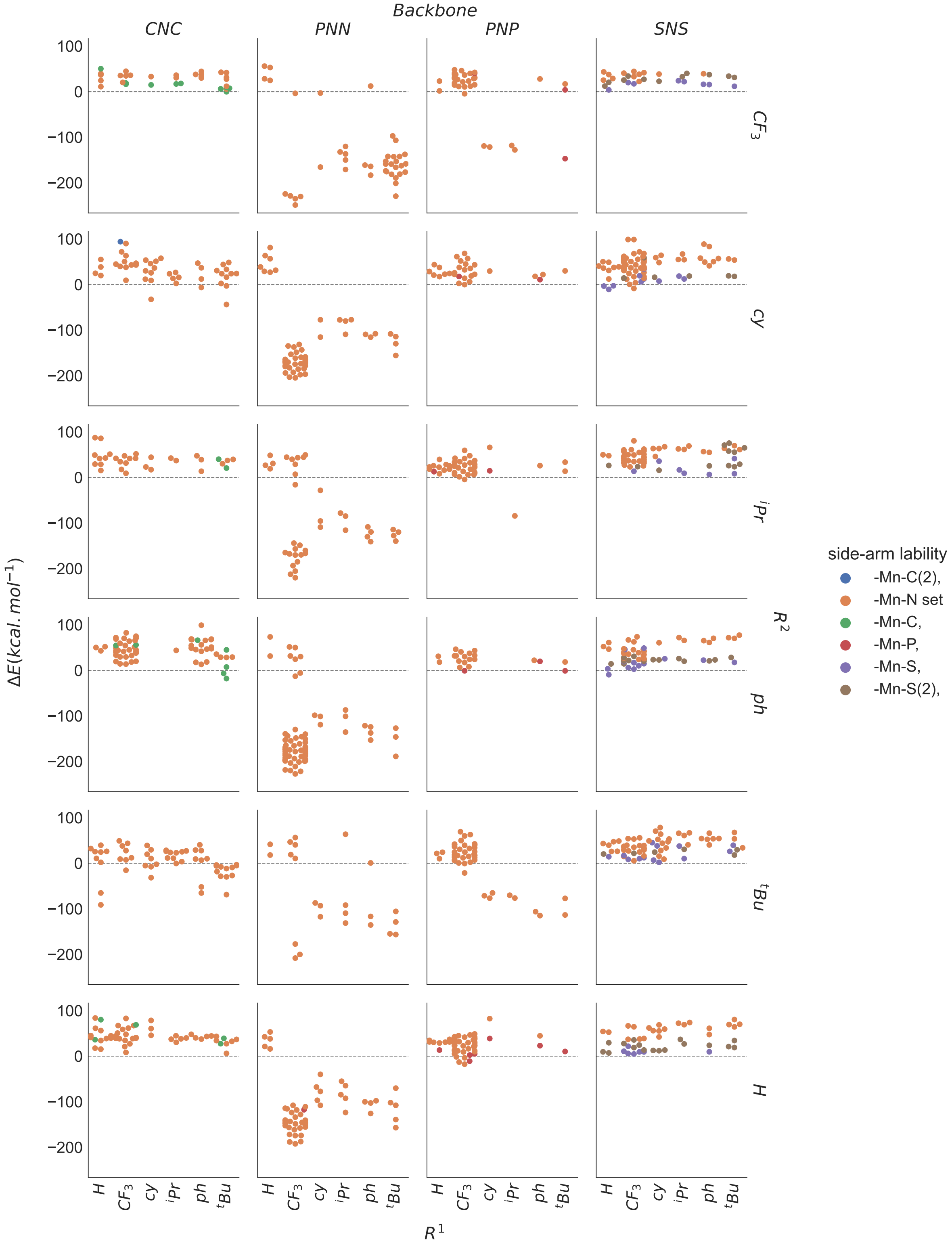

Figure S16: Decoordination of the donor atoms in the Mn(I) complexes with CNC, PNN, PNP and SNS backbones within [-250 , 100 kcal.mol^{-1}] ΔE from reference structures. Structures are colored based on the type of the donor atom dissociated as presented in the legend.

S4. Supplementary Tables

Table S2: Exploration data for structures where decoordination of the central Mn-C bond has been observed: Structural (R_1, R_2, adduct, backbone) and reactive (ΔE(kcal mol^{-1}), '-Mn-C' side-arm lability, coordination number of Mn (CN)) features are listed for explored structures. Rows for structures in [0,25] and [-40, 0] kcal mol^{-1} are respectively colored in green and orange.

	ID	CN	ΔE(kcal mol^{-1})	Adduct	R_1	R_2	backbone	side-arm lability
1	Br-CNC-CF_3-cy-2	6	94.16	Br	CF_3	cy	CNC	-Mn-C(2)
2	Br-CNC-CF_3-cy-3	5	89.96	Br	CF_3	cy	CNC	-Mn-N, -Mn-C(2)
3	Br-CNC-cy-^{t}Bu-2	5	39.12	Br	cy	^{t}Bu	CNC	-Mn-N, -Mn-C
4	Br-CNC-^{i}Pr-CF_3-2	5	16.89	Br	^{i}Pr	CF_3	CNC	-Mn-C
5	Br-CNC-^{i}Pr-CF_3-3	6	18.04	Br	^{i}Pr	CF_3	CNC	-Mn-C
6	Br-CNC-ph-ph-2	5	41.79	Br	ph	ph	CNC	-Mn-N, -Mn-C(2)
7	Br-CNC-ph-ph-3	5	68.8	Br	ph	ph	CNC	-Mn-N, -Mn-C(2)
8	Br-CNC-ph-ph-4	4	67.38	Br	ph	ph	CNC	-Mn-N, -Mn-C(2)
9	Br-CNC-ph-ph-5	5	99.08	Br	ph	ph	CNC	-Mn-N, -Mn-C(2)
10	Br-CNC-^{t}Bu-CF_3-3	6	6.11	Br	^{t}Bu	CF_3	CNC	-Mn-C
11	Br-CNC-^{t}Bu-CF_3-4	5	7.44	Br	^{t}Bu	CF_3	CNC	-Mn-C
12	Br-CNC-^{t}Bu-CF_3-5	5	41.77	Br	^{t}Bu	CF_3	CNC	-Mn-N, -Mn-C
13	Br-CNC-^{t}Bu-CF_3-6	4	42.5	Br	^{t}Bu	CF_3	CNC	-Mn-N, -Mn-C
14	Br-CNC-^{t}Bu-CF_3-7	5	31.35	Br	^{t}Bu	CF_3	CNC	-Mn-N, -Mn-C
15	Br-CNC-^{t}Bu-CF_3-8	6	27.43	Br	^{t}Bu	CF_3	CNC	-Mn-N, -Mn-C
16	Br-CNC-^{t}Bu-cy-3	4	-2.93	Br	^{t}Bu	cy	CNC	-Mn-N, -Mn-C
17	Br-CNC-^{t}Bu-^{i}Pr-3	5	20.53	Br	^{t}Bu	^{i}Pr	CNC	-Mn-C
18	Br-CNC-^{t}Bu-^{t}Bu-3	5	-26.92	Br	^{t}Bu	^{t}Bu	CNC	-Mn-N, -Mn-C
19	Br-CNC-^{t}Bu-2	5	27.43	Br	^{t}Bu	H	CNC	-Mn-C
20	Br-CNC-^{t}Bu-3	5	43.31	Br	^{t}Bu	H	CNC	-Mn-N, -Mn-C
21	Br-CNC-^{t}Bu-4	5	36.82	Br	^{t}Bu	H	CNC	-Mn-N, -Mn-C
22	O^{t}Bu-CNC-CF_3-CF_3-5	5	16.33	O^{t}Bu	CF_3	CF_3	CNC	-Mn-C
23	O^{t}Bu-CNC-CF_3-CF_3-6	4	44.73	O^{t}Bu	CF_3	CF_3	CNC	-Mn-N, -Mn-C
24	O^{t}Bu-CNC-CF_3-cy-5	4	42.97	O^{t}Bu	CF_3	cy	CNC	-Mn-N, -Mn-C
25	O^{t}Bu-CNC-CF_3-cy-6	4	71.86	O^{t}Bu	CF_3	cy	CNC	-Mn-N, -Mn-C(2)
26	O^{t}Bu-CNC-CF_3-ph-5	5	65.67	O^{t}Bu	CF_3	ph	CNC	-Mn-N, -Mn-C
27	O^{t}Bu-CNC-CF_3-ph-6	6	70.68	O^{t}Bu	CF_3	ph	CNC	-Mn-N, -Mn-C
28	O^{t}Bu-CNC-CF_3-ph-10	6	64.03	O^{t}Bu	CF_3	ph	CNC	-Mn-N, -Mn-C
29	O^{t}Bu-CNC-CF_3-ph-11	5	71.77	O^{t}Bu	CF_3	ph	CNC	-Mn-N, -Mn-C

30	O^{t}Bu-CNC-CF$_3$-ph-12	5	59.34	O^{t}Bu	CF$_3$	ph	CNC	-Mn-N, -Mn-C(2)
31	O^{t}Bu-CNC-CF$_3$-ph-13	5	54.52	O^{t}Bu	CF$_3$	ph	CNC	-Mn-N, -Mn-C
32	O^{t}Bu-CNC-CF$_3$-ph-14	5	14.3	O^{t}Bu	CF$_3$	ph	CNC	-Mn-N, -Mn-C
33	O^{t}Bu-CNC-CF$_3$-ph-15	4	29.41	O^{t}Bu	CF$_3$	ph	CNC	-Mn-N, -Mn-C
34	O^{t}Bu-CNC-CF$_3$-ph-16	5	29.48	O^{t}Bu	CF$_3$	ph	CNC	-Mn-N, -Mn-C
35	O^{t}Bu-CNC-CF$_3$-ph-17	5	18.32	O^{t}Bu	CF$_3$	ph	CNC	-Mn-N, -Mn-C
36	O^{t}Bu-CNC-CF$_3$-ph-18	4	33.52	O^{t}Bu	CF$_3$	ph	CNC	-Mn-N, -Mn-C(2)
37	O^{t}Bu-CNC-CF$_3$-ph-19	6	54.55	O^{t}Bu	CF$_3$	ph	CNC	-Mn-C
38	O^{t}Bu-CNC-CF$_3$-^{t}Bu-6	4	38.07	O^{t}Bu	CF$_3$	^{t}Bu	CNC	-Mn-N, -Mn-C
39	O^{t}Bu-CNC-CF$_3$-^{t}Bu-7	5	49.16	O^{t}Bu	CF$_3$	^{t}Bu	CNC	-Mn-N, -Mn-C
40	O^{t}Bu-CNC-CF$_3$-^{t}Bu-8	4	43.77	O^{t}Bu	CF$_3$	^{t}Bu	CNC	-Mn-N, -Mn-C(2)
41	O^{t}Bu-CNC-CF$_3$-3	4	27.08	O^{t}Bu	CF$_3$	H	CNC	-Mn-N, -Mn-C
42	O^{t}Bu-CNC-CF$_3$-9	6	82.83	O^{t}Bu	CF$_3$	H	CNC	-Mn-N, -Mn-C
43	O^{t}Bu-CNC-CF$_3$-10	5	66.98	O^{t}Bu	CF$_3$	H	CNC	-Mn-N, -Mn-C
44	O^{t}Bu-CNC-cy-CF$_3$-8	5	14.78	O^{t}Bu	Cy	CF$_3$	CNC	-Mn-C
45	O^{t}Bu-CNC-cy-5	5	78.44	O^{t}Bu	Cy	H	CNC	-Mn-N, -Mn-C(2)
46	O^{t}Bu-CNC-H-CF$_3$-5	5	50.35	O^{t}Bu	H	CF$_3$	CNC	-Mn-C
47	O^{t}Bu-CNC-H-CF$_3$-6	4	36.77	O^{t}Bu	H	CF$_3$	CNC	-Mn-N, -Mn-C
48	O^{t}Bu-CNC-H-cy-3	4	55.07	O^{t}Bu	H	Cy	CNC	-Mn-N, -Mn-C
49	O^{t}Bu-CNC-H-^{i}Pr-5	4	49.11	O^{t}Bu	H	^{i}Pr	CNC	-Mn-N, -Mn-C
50	O^{t}Bu-CNC-H-^{t}Bu-2	4	26.18	O^{t}Bu	H	^{t}Bu	CNC	-Mn-N, -Mn-C
51	O^{t}Bu-CNC-H-2	5	36.18	O^{t}Bu	H	H	CNC	-Mn-C
52	O^{t}Bu-CNC-^{i}Pr-CF$_3$-5	4	36.12	O^{t}Bu	^{i}Pr	CF$_3$	CNC	-Mn-N, -Mn-C
53	O^{t}Bu-CNC-^{i}Pr-^{t}Bu-2	4	11.57	O^{t}Bu	^{i}Pr	^{t}Bu	CNC	-Mn-N, -Mn-C
54	O^{t}Bu-CNC-^{i}Pr-^{t}Bu-5	4	23.63	O^{t}Bu	^{i}Pr	^{t}Bu	CNC	-Mn-N, -Mn-C
55	O^{t}Bu-CNC-^{i}Pr-^{t}Bu-8	5	27.07	O^{t}Bu	^{i}Pr	^{t}Bu	CNC	-Mn-N, -Mn-C
56	O^{t}Bu-CNC-^{i}Pr-4	5	44.79	O^{t}Bu	^{i}Pr	H	CNC	-Mn-N, -Mn-C
57	O^{t}Bu-CNC-^{i}Pr-5	4	39.94	O^{t}Bu	^{i}Pr	H	CNC	-Mn-N, -Mn-C
58	O^{t}Bu-CNC-ph-CF$_3$-4	4	29.98	O^{t}Bu	Ph	CF$_3$	CNC	-Mn-N, -Mn-C
59	O^{t}Bu-CNC-ph-CF$_3$-5	4	32.19	O^{t}Bu	Ph	CF$_3$	CNC	-Mn-N, -Mn-C
60	O^{t}Bu-CNC-ph-CF$_3$-6	4	44.46	O^{t}Bu	Ph	CF$_3$	CNC	-Mn-N, -Mn-C
61	O^{t}Bu-CNC-ph-^{t}Bu-4	4	40.41	O^{t}Bu	Ph	^{t}Bu	CNC	-Mn-N, -Mn-C
62	O^{t}Bu-CNC-^{t}Bu-CF$_3$-2	4	5.81	O^{t}Bu	^{t}Bu	CF$_3$	CNC	-Mn-N, -Mn-C
63	O^{t}Bu-CNC-^{t}Bu-CF$_3$-3	5	-0.28	O^{t}Bu	^{t}Bu	CF$_3$	CNC	-Mn-C
64	O^{t}Bu-CNC-^{t}Bu-ph-2	5	-18.05	O^{t}Bu	^{t}Bu	Ph	CNC	-Mn-C

65	O^tBu-CNC-^tBu-^tBu-3	4	-18.33	O^tBu	^tBu	^tBu	CNC	-Mn-N, -Mn-C
66	O^tBu-CNC-^tBu-^tBu-4	4	-4.24	O^tBu	^tBu	^tBu	CNC	-Mn-N, -Mn-C
67	O^tBu-CNC-^tBu-5	5	39.06	O^tBu	^tBu	H	CNC	-Mn-C
68	OCH_3-CNC-CF_3-CF_3-11	5	18.71	OCH_3	CF_3	CF_3	CNC	-Mn-C
69	OCH_3-CNC-CF_3-^iPr-3	4	31.61	OCH_3	CF_3	^iPr	CNC	-Mn-N, -Mn-C
70	OCH_3-CNC-CF_3-^iPr-9	4	47.4	OCH_3	CF_3	^iPr	CNC	-Mn-N, -Mn-C
71	OCH_3-CNC-CF_3-ph-8	6	68.73	OCH_3	CF_3	ph	CNC	-Mn-N, -Mn-C
72	OCH_3-CNC-CF_3-ph-9	5	74.91	OCH_3	CF_3	ph	CNC	-Mn-N, -Mn-C
73	OCH_3-CNC-CF_3-ph-10	5	55.84	OCH_3	CF_3	ph	CNC	-Mn-C
74	OCH_3-CNC-CF_3-6	6	58.67	OCH_3	CF_3	H	CNC	-Mn-N, -Mn-C
75	OCH_3-CNC-CF_3-7	5	61.59	OCH_3	CF_3	H	CNC	-Mn-N, -Mn-C
76	OCH_3-CNC-CF_3-8	4	67.13	OCH_3	CF_3	H	CNC	-Mn-N, -Mn-C
77	OCH_3-CNC-CF_3-9	6	40.72	OCH_3	CF_3	H	CNC	-Mn-N, -Mn-C
78	OCH_3-CNC-CF_3-10	6	47.97	OCH_3	CF_3	H	CNC	-Mn-N, -Mn-C
79	OCH_3-CNC-CF_3-11	5	40.32	OCH_3	CF_3	H	CNC	-Mn-N, -Mn-C(2)
80	OCH_3-CNC-CF_3-12	6	44.51	OCH_3	CF_3	H	CNC	-Mn-N, -Mn-C
81	OCH_3-CNC-H-ph-5	5	51.96	OCH_3	H	ph	CNC	-Mn-N, -Mn-C
82	OCH_3-CNC-H-5	4	56.13	OCH_3	H	H	CNC	-Mn-N, -Mn-C
83	OCH_3-CNC-ph-ph-6	6	65.67	OCH_3	Ph	ph	CNC	-Mn-N, -Mn-C
84	OCH_3-CNC-ph-ph-7	6	68.51	OCH_3	ph	ph	CNC	-Mn-N, -Mn-C
85	OCH_3-CNC-^tBu-CF_3-4	5	9.15	OCH_3	^tBu	CF_3	CNC	-Mn-C
86	OCH_3-CNC-^tBu-CF_3-5	5	3.69	OCH_3	^tBu	CF_3	CNC	-Mn-C
87	OCH_3-CNC-^tBu-CF_3-6	4	11.77	OCH_3	^tBu	CF_3	CNC	-Mn-N, -Mn-C
88	OCH_3-CNC-^tBu-ph-2	5	7.27	OCH_3	^tBu	ph	CNC	-Mn-C
89	OH-CNC-CF_3-cy-5	5	63.25	OH	CF_3	cy	CNC	-Mn-N, -Mn-C
90	OH-CNC-CF_3-ph-3	5	19.31	OH	CF_3	ph	CNC	-Mn-N, -Mn-C
91	OH-CNC-CF_3-ph-4	5	32.51	OH	CF_3	ph	CNC	-Mn-N, -Mn-C
92	OH-CNC-CF_3-ph-11	5	70.2	OH	CF_3	ph	CNC	-Mn-N, -Mn-C
93	OH-CNC-CF_3-ph-12	6	82.39	OH	CF_3	ph	CNC	-Mn-N, -Mn-C
94	OH-CNC-CF_3-5	6	68.73	OH	CF_3	H	CNC	-Mn-C
95	OH-CNC-H-^iPr-6	6	87.29	OH	H	^iPr	CNC	-Mn-N, -Mn-C
96	OH-CNC-H-^iPr-7	5	111.13	OH	H	^iPr	CNC	-Mn-N, -Mn-C(2)
97	OH-CNC-H-^iPr-8	7	85.81	OH	H	^iPr	CNC	-Mn-N, -Mn-C
98	OH-CNC-H-^iPr-9	6	105.24	OH	H	^iPr	CNC	-Mn-N, -Mn-C
99	OH-CNC-H-6	5	80.06	OH	H	H	CNC	-Mn-C

100	OH-CNC-ph-ph-9	5	66.12	OH	ph	ph	CNC	-Mn-C
101	OH-CNC-ph-ph-10	5	67.68	OH	ph	ph	CNC	-Mn-N, -Mn-C
102	OH-CNC-ph-ph-11	4	67.99	OH	ph	ph	CNC	-Mn-N, -Mn-C
103	OH-CNC-^{t}Bu-^{i}Pr-5	6	39.97	OH	^{t}Bu	^{i}Pr	CNC	-Mn-C
104	OH-CNC-^{t}Bu-^{i}Pr-6	4	39.58	OH	^{t}Bu	^{i}Pr	CNC	-Mn-N, -Mn-C
105	OH-CNC-^{t}Bu-^{i}Pr-7	5	37.25	OH	^{t}Bu	^{i}Pr	CNC	-Mn-N, -Mn-C
106	OH-CNC-^{t}Bu-ph-5	5	44.95	OH	^{t}Bu	ph	CNC	-Mn-C
107	OH-CNC-^{t}Bu-ph-6	5	-6.63	OH	^{t}Bu	ph	CNC	-Mn-C

Table S3: Exploration results for structures with "-N-H, +H-O interaction"

	ID	CN	ΔE(kcal mol^{-1})	Adduct	R_1	R_2	backbone	interaction
1	OCH_3-CNC-H-CF_3-3	5	-0.36	OCH_3	H	CF_3	CNC	-N-H, +H-O
2	OH-CNC-CF_3-CF_3-2	6	-12.93	OH	CF_3	CF_3	CNC	-N-H, +H-O
3	OH-CNC-CF_3-CF_3-3	6	-17.89	OH	CF_3	CF_3	CNC	-N-H, +H-O, +Mn-F
4	OH-CNC-cy-CF_3-2	5	-25.09	OH	cy	CF_3	CNC	-N-H, +H-O
5	OH-CNC-cy-CF_3-3	6	-25.89	OH	cy	CF_3	CNC	-N-H, +H-O, +Mn-F
6	OH-CNC-H-CF_3-2	6	-13.07	OH	H	CF_3	CNC	-N-H, +H-O
7	OH-CNC-H-CF_3-3	5	-10.72	OH	H	CF_3	CNC	-N-H, +H-O
8	OH-CNC-H-10	6	-3.46	OH	H	H	CNC	-N-H, +H-O
9	OH-CNC-^{i}Pr-CF_3-2	6	-14.08	OH	^{i}Pr	CF_3	CNC	-N-H, +H-O
10	OH-CNC-^{i}Pr-CF_3-3	6	-12.42	OH	^{i}Pr	CF_3	CNC	-N-H, +H-O, +Mn-F
11	OH-CNC-^{i}Pr-CF_3-5	5	-6.79	OH	^{i}Pr	CF_3	CNC	-N-H, +H-O
12	OH-CNC-ph-CF_3-2	6	-18.51	OH	ph	CF_3	CNC	-N-H, +H-O
13	OH-CNC-ph-CF_3-3	5	-19.82	OH	ph	CF_3	CNC	-N-H, +H-O
14	OH-CNC-^{t}Bu-CF_3-2	6	-17.88	OH	^{t}Bu	CF_3	CNC	-N-H, +H-O

Table S4: Nucleophilic (+C-O) interactions observed for CNC structures with different R1-R2-Adduct combinations

	ID	CN	ΔE(kcal mol^{-1})	Adduct	R_1	R_2	backbone	Interaction
1	OCH_3-CNC-CF_3-^{t}Bu-2	5	-4.4	OCH_3	CNC	CF_3	^{t}Bu	+C-O
2	OCH_3-CNC-cy-^{i}Pr-2	5	-0.96	OCH_3	CNC	cy	^{i}Pr	+C-O
3	OCH_3-CNC-^{i}Pr-cy-2	5	-13.84	OCH_3	CNC	^{i}Pr	Cy	+C-O
4	OCH_3-CNC-^{t}Bu-^{i}Pr-2	5	-16.76	OCH_3	CNC	^{t}Bu	^{i}Pr	+C-O

5	OH-CNC-CF_3-^{t}Bu-6	5	-5.9	OH	CNC	CF_3	^{t}Bu	+C-O
6	OH-CNC-^{i}Pr-^{i}Pr-2	5	-11.37	OH	CNC	^{i}Pr	^{i}Pr	+C-O
7	OH-CNC-ph-^{i}Pr-2	5	-3.9	OH	CNC	Ph	^{i}Pr	+C-O
8	OH-CNC-ph-^{i}Pr-3	6	-4.96	OH	CNC	Ph	^{i}Pr	+C-O
9	OH-CNC-^{t}Bu-cy-2	6	-9.63	OH	CNC	^{t}Bu	Cy	+C-O
10	OH-CNC-^{t}Bu-^{t}Bu-3	5	-10.75	OH	CNC	^{t}Bu	^{t}Bu	+C-O
11	OH-CNC-^{t}Bu-2	6	-3.19	OH	CNC	^{t}Bu	H	+C-O
12	O^{t}Bu-PNP-CF_3-^{t}Bu-3	5	-6.6	O^{t}Bu	PNP	CF_3	^{t}Bu	+C-O
13	OCH_3-CNC-^{t}Bu-^{t}Bu-2	6	-11.2	OCH_3	CNC	^{t}Bu	^{t}Bu	+C-O, +Mn-H
14	OH-CNC-CF_3-^{t}Bu-2	6	-9.19	OH	CNC	CF_3	^{t}Bu	+C-O, +Mn-H
15	OH-CNC-cy-^{t}Bu-3	6	-3.14	OH	CNC	cy	^{t}Bu	+C-O, +Mn-H
16	OH-CNC-^{i}Pr-^{t}Bu-2	6	-12.06	OH	CNC	^{i}Pr	^{t}Bu	+C-O, +Mn-H
17	OH-CNC-ph-^{t}Bu-2	6	-7.18	OH	CNC	ph	^{t}Bu	+C-O, +Mn-H
18	OH-CNC-^{t}Bu-^{t}Bu-2	6	-5.38	OH	CNC	^{t}Bu	^{t}Bu	+C-O, +Mn-H
19	O^{t}Bu-PNP-CF_3-^{t}Bu-4	6	-11.54	O^{t}Bu	PNP	CF_3	^{t}Bu	+C-O, +Mn-H
20	OH-CNC-^{t}Bu-ph-6	5	-6.63	OH	CNC	^{t}Bu	Ph	-Mn-C, +C-O, +C-H, -O-H
21	O^{t}Bu-CNC-cy-^{t}Bu-3	5	-2.23	O^{t}Bu	CNC	Cy	^{t}Bu	-Mn-N, +C-O
22	O^{t}Bu-CNC-cy-^{t}Bu-5	4	-7.64	O^{t}Bu	CNC	Cy	^{t}Bu	-Mn-N, +C-O
23	OCH_3-CNC-^{t}Bu-^{t}Bu-5	4	-3.94	OCH_3	CNC	^{t}Bu	^{t}Bu	-Mn-N, +C-O
24	OH-CNC-^{i}Pr-^{t}Bu-6	4	-0.43	OH	CNC	^{i}Pr	^{t}Bu	-Mn-N, +C-O
25	O^{t}Bu-CNC-cy-^{t}Bu-4	6	-5.15	O^{t}Bu	CNC	Cy	^{t}Bu	-Mn-N, +C-O, +Mn-H
26	O^{t}Bu-CNC-^{t}Bu-^{t}Bu-5	5	-5.83	O^{t}Bu	CNC	^{t}Bu	^{t}Bu	-Mn-N, +C-O, +Mn-H
27	OCH_3-CNC-^{t}Bu-^{t}Bu-3	5	-9.18	OCH_3	CNC	^{t}Bu	^{t}Bu	-Mn-N, +C-O, +Mn-H
28	OCH_3-CNC-^{t}Bu-^{t}Bu-4	5	-3.28	OCH_3	CNC	^{t}Bu	^{t}Bu	-Mn-N, +C-O, +Mn-H
29	OCH_3-CNC-^{t}Bu-^{t}Bu-7	6	-5.06	OCH_3	CNC	^{t}Bu	^{t}Bu	-Mn-N, +C-O, +Mn-H
30	OH-CNC-^{t}Bu-^{t}Bu-4	5	-3.9	OH	CNC	^{t}Bu	^{t}Bu	-Mn-N, +C-O, +Mn-H
31	OH-CNC-^{t}Bu-^{t}Bu-5	5	-11.72	OH	CNC	^{t}Bu	^{t}Bu	-Mn-N, +C-O, +Mn-H
32	O^{t}Bu-CNC-^{t}Bu-^{t}Bu-6	6	-7.83	O^{t}Bu	CNC	^{t}Bu	^{t}Bu	-Mn-N, +C-O, +Mn-H, +Mn-H

Table S5: Nucleophilic (+C-C) interactions observed for CNC structures with different R1-R2-Adduct combinations

	ID	CN	ΔE(kcal mol^{-1})	Adduct	R_1	R_2	backbone	Interaction
1	Br-CNC-^{t}Bu-cy-3	4	-2.93	Br	CNC	^{t}Bu	cy	-Mn-N, -Mn-C, +C-C
2	Br-CNC-^{t}Bu-^{t}Bu-3	5	-26.92	Br	CNC	^{t}Bu	^{t}Bu	-Mn-N, -Mn-C, +C-C, +Mn-H
3	O^{t}Bu-CNC-^{t}Bu-^{t}Bu-4	4	-4.24	O^{t}Bu	CNC	^{t}Bu	^{t}Bu	-Mn-N, -Mn-C, +C-C

Table S6: Formation of Manganese carbonyl trifluoromethyl complexes with PNP

	ID	CN	ΔE(kcal mol^{-1})	Adduct	R_1	R_2	backbone	Interaction
1	O^tBu-PNP-CF_3-4	6	-39.2	O^tBu	PNP	CF_3	H	+Mn-C, -P-C, +P-O
2	OCH_3-PNP-CF_3-^{i}Pr-4	6	-9.56	OCH_3	PNP	CF_3	^{i}Pr	+Mn-C, -P-C, +P-O
3	OCH_3-PNP-CF_3-^{t}Bu-3	6	-0.83	OCH_3	PNP	CF_3	^{t}Bu	+Mn-C, -P-C, +P-O
4	OCH_3-PNP-CF_3-4	6	-23.05	OCH_3	PNP	CF_3	H	+Mn-C, -P-C, +P-O
5	OH-PNP-CF_3-CF_3-7	6	-9.43	OH	PNP	CF_3	CF_3	+Mn-C, -P-C, +P-O
6	OH-PNP-CF_3-cy-5	6	-8.18	OH	PNP	CF_3	Cy	+Mn-C, -P-C, +P-O
7	OH-PNP-CF_3-ph-7	6	-17.16	OH	PNP	CF_3	ph	+Mn-C, -P-C, +P-O
8	O^tBu-PNP-CF_3-2	6	-26.52	O^tBu	PNP	CF_3	H	+P-O
9	OCH_3-PNP-CF_3-2	6	-11.81	OCH_3	PNP	CF_3	H	+P-O
10	OH-PNP-CF_3-ph-3	6	-3.32	OH	PNP	CF_3	ph	+P-O
11	OH-PNP-CF_3-2	6	-10.84	OH	PNP	CF_3	H	+P-O
12	O^tBu-PNP-CF_3-3	6	-6.72	O^tBu	PNP	CF_3	H	+P-O, +Mn-F
13	OH-PNP-CF_3-15	5	-3.58	OH	PNP	CF_3	H	-Mn-N, +Mn-C, +N-H, -P-C, +P-O, -O-H
14	O^tBu-PNP-CF_3-5	5	-13.03	O^tBu	PNP	CF_3	H	-Mn-N, +Mn-C, +N-P, -P-C, +P-O
15	OH-PNP-CF_3-^{i}Pr-9	6	-4.46	OH	PNP	CF_3	^{i}Pr	-Mn-N, +Mn-C, +N-P, -P-C, +P-O
16	O^tBu-PNP-CF_3-CF_3-3	5	-4.63	O^tBu	PNP	CF_3	CF_3	-Mn-N, +Mn-C, -P-C, +P-O
17	O^tBu-PNP-CF_3-8	5	-17.65	O^tBu	PNP	CF_3	H	-Mn-N, +Mn-C, -P-C, +P-O
18	OH-PNP-CF_3-^{t}Bu-13	5	-0.6	OH	PNP	CF_3	^{t}Bu	-Mn-N, +Mn-C, -P-C, +P-O
19	OH-PNP-CF_3-21	5	-1.55	OH	PNP	CF_3	H	-Mn-N, -Mn-P, +Mn-C, +N-H, -P-C, +P-O, -O-H
20	OH-PNP-CF_3-cy-2	6	-0.19	OH	PNP	CF_3	cy	-Mn-N, -Mn-P, +Mn-C, +N-H, -P-C, +P-O, -O-H, +Mn-F
21	O^tBu-PNP-CF_3-6	5	-10.97	O^tBu	PNP	CF_3	H	-Mn-P, +Mn-C, -P-C, +P-O

Table S7: Summary of data on reactivity exploration for species lower energies than their respective reference structures

	ID	CN	ΔE(kcal mol^{-1})	Adduct	R_1	R_2	backbone	Interaction
1	Br-CNC-^{t}Bu-cy-2	5	-43.77	Br	^{t}Bu	cy	CNC	-Mn-N
2	Br-CNC-^{t}Bu-cy-3	4	-2.93	Br	^{t}Bu	cy	CNC	-Mn-N, -Mn-C, +C-C
3	Br-CNC-^{t}Bu-^{t}Bu-2	5	-68.79	Br	^{t}Bu	^{t}Bu	CNC	-Mn-N
4	Br-CNC-^{t}Bu-^{t}Bu-3	5	-26.92	Br	^{t}Bu	^{t}Bu	CNC	-Mn-N, -Mn-C, +C-C, +Mn-H
5	O^tBu-CNC-cy-cy-2	5	-32.46	O^tBu	cy	cy	CNC	-Mn-N
6	O^tBu-CNC-cy-^{t}Bu-2	5	-31.68	O^tBu	cy	^{t}Bu	CNC	-Mn-N
7	O^tBu-CNC-cy-^{t}Bu-3	5	-2.23	O^tBu	cy	^{t}Bu	CNC	-Mn-N, +C-O, +Mn-O, +Mn-O
8	O^tBu-CNC-cy-^{t}Bu-4	6	-5.15	O^tBu	cy	^{t}Bu	CNC	-Mn-N, +C-O, +Mn-H

9	O^tBu-CNC-cy-^tBu-5	4	-7.64	O^tBu	cy	^tBu	CNC	-Mn-N, +C-O, +Mn-O
10	O^tBu-CNC-ph-cy-2	5	-6.4	O^tBu	ph	cy	CNC	-Mn-N
11	O^tBu-CNC-^tBu-CF_3-3	5	-0.28	O^tBu	^tBu	CF_3	CNC	-Mn-C, -N-H, +C-H
12	O^tBu-CNC-^tBu-ph-2	5	-18.05	O^tBu	^tBu	ph	CNC	-Mn-C
13	O^tBu-CNC-^tBu-^tBu-2	5	-29.85	O^tBu	^tBu	^tBu	CNC	-Mn-N
14	O^tBu-CNC-^tBu-^tBu-3	4	-18.33	O^tBu	^tBu	^tBu	CNC	-Mn-N, -Mn-C
15	O^tBu-CNC-^tBu-^tBu-4	4	-4.24	O^tBu	^tBu	^tBu	CNC	-Mn-N, -Mn-C, +C-C
16	O^tBu-CNC-^tBu-^tBu-5	5	-5.83	O^tBu	^tBu	^tBu	CNC	-Mn-N, +C-O, +Mn-H, +Mn-O
17	O^tBu-CNC-^tBu-^tBu-6	6	-7.83	O^tBu	^tBu	^tBu	CNC	-Mn-N, +C-O, +Mn-H, +Mn-H, +Mn-O
18	OCH_3-CNC-CF_3-^tBu-2	5	-4.4	OCH_3	CF_3	^tBu	CNC	+C-O, +Mn-O
19	OCH_3-CNC-cy-^iPr-2	5	-0.96	OCH_3	cy	^iPr	CNC	+C-O, +Mn-O
20	OCH_3-CNC-cy-ph-2	5	-53.74	OCH_3	cy	ph	CNC	+C-O, +Mn-O
21	OCH_3-CNC-H-CF_3-3	5	-0.36	OCH_3	H	CF_3	CNC	-N-H, +H-O, +Mn-O
22	OCH_3-CNC-H-^tBu-2	5	-91.33	OCH_3	H	^tBu	CNC	-Mn-N
23	OCH_3-CNC-H-^tBu-3	5	-65.19	OCH_3	H	^tBu	CNC	-Mn-N, +C-O, +Mn-O, +Mn-O
24	OCH_3-CNC-^iPr-cy-2	5	-13.84	OCH_3	^iPr	cy	CNC	+C-O, +Mn-O
25	OCH_3-CNC-ph-^tBu-2	6	-82.1	OCH_3	ph	^tBu	CNC	+C-O, +Mn-H, +Mn-O
26	OCH_3-CNC-ph-^tBu-3	5	-51.97	OCH_3	ph	^tBu	CNC	-Mn-N, +Mn-C, +C-O, +Mn-O
27	OCH_3-CNC-ph-^tBu-4	6	-65.3	OCH_3	ph	^tBu	CNC	-Mn-N, +C-O, +Mn-O, +Mn-H, +Mn-O
28	OCH_3-CNC-^tBu-^iPr-2	5	-16.76	OCH_3	^tBu	^iPr	CNC	+C-O, +Mn-O
29	OCH_3-CNC-^tBu-^tBu-2	6	-11.2	OCH_3	^tBu	^tBu	CNC	+C-O, +Mn-H, +Mn-O
30	OCH_3-CNC-^tBu-^tBu-3	5	-9.18	OCH_3	^tBu	^tBu	CNC	-Mn-N, +C-O, +Mn-H, +Mn-O
31	OCH_3-CNC-^tBu-^tBu-4	5	-3.28	OCH_3	^tBu	^tBu	CNC	-Mn-N, +C-O, +Mn-H, +Mn-O
32	OCH_3-CNC-^tBu-^tBu-5	4	-3.94	OCH_3	^tBu	^tBu	CNC	-Mn-N, +C-O, +Mn-O
33	OCH_3-CNC-^tBu-^tBu-6	5	-28.57	OCH_3	^tBu	^tBu	CNC	-Mn-N
34	OCH_3-CNC-^tBu-^tBu-7	6	-5.06	OCH_3	^tBu	^tBu	CNC	-Mn-N, +C-O, +Mn-O, +Mn-H, +Mn-O
35	OH-CNC-CF_3-CF_3-2	6	-12.93	OH	CF_3	CF_3	CNC	-N-H, +H-O
36	OH-CNC-CF_3-CF_3-3	6	-17.89	OH	CF_3	CF_3	CNC	-N-H, +H-O, +Mn-F, +Mn-O
37	OH-CNC-CF_3-^tBu-2	6	-9.19	OH	CF_3	^tBu	CNC	+C-O, +Mn-H, +Mn-O
38	OH-CNC-CF_3-^tBu-3	5	-15.35	OH	CF_3	^tBu	CNC	-Mn-N
39	OH-CNC-CF_3-^tBu-6	5	-5.9	OH	CF_3	^tBu	CNC	+C-O, +Mn-O
40	OH-CNC-cy-CF_3-2	5	-25.09	OH	cy	CF_3	CNC	-N-H, +H-O, +Mn-O
41	OH-CNC-cy-CF_3-3	6	-25.89	OH	cy	CF_3	CNC	-N-H, +H-O, +Mn-F, +Mn-O
42	OH-CNC-cy-^tBu-3	6	-3.14	OH	cy	^tBu	CNC	+C-O, +Mn-H, +Mn-O
43	OH-CNC-H-CF_3-2	6	-13.07	OH	H	CF_3	CNC	-N-H, +H-O
44	OH-CNC-H-CF_3-3	5	-10.72	OH	H	CF_3	CNC	-N-H, +H-O, +Mn-O

45	OH-CNC-H-10	6	-3.46	OH	H	H	CNC	-N-H, +H-O
46	OH-CNC-^{i}Pr-CF_3-2	6	-14.08	OH	^{i}Pr	CF_3	CNC	-N-H, +H-O
47	OH-CNC-^{i}Pr-CF_3-3	6	-12.42	OH	^{i}Pr	CF_3	CNC	-N-H, +H-O, +Mn-F, +Mn-O
48	OH-CNC-^{i}Pr-CF_3-5	5	-6.79	OH	^{i}Pr	CF_3	CNC	-N-H, +H-O, +Mn-O
49	OH-CNC-^{i}Pr-^{i}Pr-2	5	-11.37	OH	^{i}Pr	^{i}Pr	CNC	+C-O, +Mn-O
50	OH-CNC-^{i}Pr-^{t}Bu-2	6	-12.06	OH	^{i}Pr	^{t}Bu	CNC	+C-O, +Mn-H, +Mn-O
51	OH-CNC-^{i}Pr-^{t}Bu-6	4	-0.43	OH	^{i}Pr	^{t}Bu	CNC	-Mn-N, +C-O, +Mn-O
52	OH-CNC-ph-CF_3-2	6	-18.51	OH	ph	CF_3	CNC	-N-H, +H-O
53	OH-CNC-ph-CF_3-3	5	-19.82	OH	ph	CF_3	CNC	-N-H, +H-O, +Mn-O
54	OH-CNC-ph-^{i}Pr-2	5	-3.9	OH	ph	^{i}Pr	CNC	+C-O, +Mn-O
55	OH-CNC-ph-^{i}Pr-3	6	-4.96	OH	ph	^{i}Pr	CNC	+C-O, +Mn-O, +Mn-O
56	OH-CNC-ph-^{t}Bu-2	6	-7.18	OH	ph	^{t}Bu	CNC	+C-O, +Mn-H, +Mn-O
57	OH-CNC-^{t}Bu-CF_3-2	6	-17.88	OH	^{t}Bu	CF_3	CNC	-N-H, +H-O
58	OH-CNC-^{t}Bu-cy-2	6	-9.63	OH	^{t}Bu	cy	CNC	+C-O, +Mn-O, +Mn-O
59	OH-CNC-^{t}Bu-ph-6	5	-6.63	OH	^{t}Bu	ph	CNC	-Mn-C, +C-O, +C-H, -O-H
60	OH-CNC-^{t}Bu-^{t}Bu-2	6	-5.38	OH	^{t}Bu	^{t}Bu	CNC	+C-O, +Mn-H, +Mn-O
61	OH-CNC-^{t}Bu-^{t}Bu-3	5	-10.75	OH	^{t}Bu	^{t}Bu	CNC	+C-O, +Mn-O
62	OH-CNC-^{t}Bu-^{t}Bu-4	5	-3.9	OH	^{t}Bu	^{t}Bu	CNC	-Mn-N, +C-O, +Mn-H, +Mn-O
63	OH-CNC-^{t}Bu-^{t}Bu-5	5	-11.72	OH	^{t}Bu	^{t}Bu	CNC	-Mn-N, +C-O, +Mn-H, +Mn-O
64	OH-CNC-^{t}Bu-2	6	-3.19	OH	^{t}Bu	H	CNC	+C-O, +Mn-O, +Mn-O
65	Br-PNN-CF_3-CF_3-2	6	-3.7	Br	CF_3	CF_3	PNN	-Mn-N, +Mn-F
66	Br-PNN-CF_3-^{i}Pr-2	5	-15.95	Br	CF_3	^{i}Pr	PNN	-Mn-N
67	Br-PNN-CF_3-ph-2	6	-12.85	Br	CF_3	ph	PNN	-Mn-N, +Mn-C
68	Br-PNN-CF_3-ph-8	5	-5.84	Br	CF_3	ph	PNN	-Mn-N
69	Br-PNN-cy-CF_3-2	4	-3.04	Br	cy	CF_3	PNN	-Mn-N, -Mn-N
70	Br-PNN-cy-cy-2	5	-77.33	Br	cy	cy	PNN	-Mn-N
71	Br-PNN-cy-2	5	-67.76	Br	cy	H	PNN	-Mn-N
72	Br-PNN-^{i}Pr-cy-2	5	-77.21	Br	^{i}Pr	cy	PNN	-Mn-N
73	Br-PNN-^{i}Pr-2	5	-64.71	Br	^{i}Pr	H	PNN	-Mn-N
74	O^{t}Bu-PNN-cy-3	4	-39.97	O^{t}Bu	cy	H	PNN	-Mn-N, -Mn-P, +Mn-H, -Mn-N, -N-H, +H-P, -C-H
75	O^{t}Bu-PNN-^{i}Pr-3	4	-55.1	O^{t}Bu	^{i}Pr	H	PNN	-Mn-N, -Mn-P, +Mn-H, -Mn-N, +C-P, -C-H
76	OCH_3-PNN-cy-^{i}Pr-2	5	-95.61	OCH_3	cy	^{i}Pr	PNN	-Mn-N
77	OCH_3-PNN-cy-^{i}Pr-3	4	-28.36	OCH_3	cy	^{i}Pr	PNN	-Mn-N, -Mn-P, +Mn-H, -Mn-N, -C-H
78	OCH_3-PNN-cy-^{t}Bu-2	5	-93.18	OCH_3	cy	^{t}Bu	PNN	-Mn-N
79	OCH_3-PNN-cy-2	5	-96.9	OCH_3	cy	H	PNN	-Mn-N
80	OCH_3-PNN-^{i}Pr-cy-2	5	-80.04	OCH_3	^{i}Pr	cy	PNN	-Mn-N

81	OCH_3-PNN-^{i}Pr-^{i}Pr-2	5	-84.9	OCH_3	^{i}Pr	^{i}Pr	PNN	-Mn-N
82	OCH_3-PNN-^{i}Pr-ph-2	5	-86.96	OCH_3	^{i}Pr	ph	PNN	-Mn-N
83	OCH_3-PNN-^{i}Pr-2	5	-92.45	OCH_3	^{i}Pr	H	PNN	-Mn-N
84	OCH_3-PNN-^{t}Bu-3	4	-70.24	OCH_3	^{t}Bu	H	PNN	-Mn-N, -Mn-P, +Mn-H, -Mn-N, +C-P, -C-H
85	OH-PNN-cy-ph-2	5	-98.58	OH	cy	ph	PNN	-Mn-N
86	OH-PNN-cy-^{t}Bu-2	5	-87.09	OH	cy	^{t}Bu	PNN	-Mn-N
87	OH-PNN-cy-2	5	-77.36	OH	cy	H	PNN	-Mn-N
88	OH-PNN-^{i}Pr-cy-2	5	-77.57	OH	^{i}Pr	cy	PNN	-Mn-N
89	OH-PNN-^{i}Pr-^{i}Pr-2	5	-78.23	OH	^{i}Pr	^{i}Pr	PNN	-Mn-N
90	OH-PNN-^{i}Pr-^{t}Bu-2	5	-91.97	OH	^{i}Pr	^{t}Bu	PNN	-Mn-N
91	OH-PNN-^{i}Pr-2	5	-84.61	OH	^{i}Pr	H	PNN	-Mn-N
92	OH-PNN-ph-2	5	-97.91	OH	ph	H	PNN	-Mn-N
93	OH-PNN-^{t}Bu-CF_3-16	6	-97.18	OH	^{t}Bu	CF_3	PNN	-Mn-N, -Mn-C, +Mn-C, +Mn-H, -Mn-N, +C-C, +C-O, -C-H, +Mn-O, +Mn-F, +Mn-O
94	Br-PNP-^{t}Bu-ph-2	5	-1.09	Br	^{t}Bu	ph	PNP	-Mn-P
95	Br-PNP-^{t}Bu-^{t}Bu-2	5	-77.09	Br	^{t}Bu	^{t}Bu	PNP	-Mn-N
96	O^{t}Bu-PNP-CF_3-CF_3-3	5	-4.63	O^{t}Bu	CF_3	CF_3	PNP	-Mn-N, +Mn-C, -P-C, +P-O, +Mn-O
97	O^{t}Bu-PNP-CF_3-ph-8	5	-0.96	O^{t}Bu	CF_3	ph	PNP	-Mn-P
98	O^{t}Bu-PNP-CF_3-^{t}Bu-2	5	-21.25	O^{t}Bu	CF_3	^{t}Bu	PNP	-Mn-N
99	O^{t}Bu-PNP-CF_3-^{t}Bu-3	5	-6.6	O^{t}Bu	CF_3	^{t}Bu	PNP	+C-O, +Mn-O
100	O^{t}Bu-PNP-CF_3-^{t}Bu-4	6	-11.54	O^{t}Bu	CF_3	^{t}Bu	PNP	+C-O, +Mn-H, +Mn-O
101	O^{t}Bu-PNP-CF_3-2	6	-26.52	O^{t}Bu	CF_3	H	PNP	+P-O
102	O^{t}Bu-PNP-CF_3-3	6	-6.72	O^{t}Bu	CF_3	H	PNP	+P-O, +Mn-F, +Mn-O
103	O^{t}Bu-PNP-CF_3-4	6	-39.2	O^{t}Bu	CF_3	H	PNP	+Mn-C, -P-C, +P-O, +Mn-O
104	O^{t}Bu-PNP-CF_3-5	5	-13.03	O^{t}Bu	CF_3	H	PNP	-Mn-N, +Mn-C, +N-P, -P-C, +P-O, +Mn-O
105	O^{t}Bu-PNP-CF_3-6	5	-10.97	O^{t}Bu	CF_3	H	PNP	-Mn-P, +Mn-C, -P-C, +P-O, +Mn-O
106	O^{t}Bu-PNP-CF_3-8	5	-17.65	O^{t}Bu	CF_3	H	PNP	-Mn-N, +Mn-C, -P-C, +P-O, +Mn-O
107	O^{t}Bu-PNP-cy-^{t}Bu-2	5	-76.6	O^{t}Bu	cy	^{t}Bu	PNP	-Mn-N
108	O^{t}Bu-PNP-^{i}Pr-^{i}Pr-2	5	-84.38	O^{t}Bu	^{i}Pr	^{i}Pr	PNP	-Mn-N
109	O^{t}Bu-PNP-^{i}Pr-^{t}Bu-2	5	-76.39	O^{t}Bu	^{i}Pr	^{t}Bu	PNP	-Mn-N
110	OCH_3-PNP-CF_3-^{i}Pr-4	6	-9.56	OCH_3	CF_3	^{i}Pr	PNP	+Mn-C, -P-C, +P-O, +Mn-O
111	OCH_3-PNP-CF_3-^{t}Bu-3	6	-0.83	OCH_3	CF_3	^{t}Bu	PNP	+Mn-C, -P-C, +P-O, +Mn-O
112	OCH_3-PNP-CF_3-2	6	-11.81	OCH_3	CF_3	H	PNP	+P-O
113	OCH_3-PNP-CF_3-4	6	-23.05	OCH_3	CF_3	H	PNP	+Mn-C, -P-C, +P-O, +Mn-O
114	OCH_3-PNP-cy-^{t}Bu-2	5	-65.1	OCH_3	cy	^{t}Bu	PNP	-Mn-N
115	OH-PNP-CF_3-CF_3-7	6	-9.43	OH	CF_3	CF_3	PNP	+Mn-C, -P-C, +P-O, +Mn-O

116	OH-PNP-CF_3-cy-2	6	-0.19	OH	CF_3	cy	PNP	-Mn-N, -Mn-P, +Mn-C, +N-H, -P-C, +P-O, -O-H, +Mn-F
117	OH-PNP-CF_3-cy-5	6	-8.18	OH	CF_3	cy	PNP	+Mn-C, -P-C, +P-O, +Mn-O
118	OH-PNP-CF_3-^{i}Pr-9	6	-4.46	OH	CF_3	^{i}Pr	PNP	-Mn-N, +Mn-C, +N-P, -P-C, +P-O
119	OH-PNP-CF_3-ph-3	6	-3.32	OH	CF_3	ph	PNP	+P-O
120	OH-PNP-CF_3-ph-7	6	-17.16	OH	CF_3	ph	PNP	+Mn-C, -P-C, +P-O, +Mn-O
121	OH-PNP-CF_3-^{t}Bu-13	5	-0.6	OH	CF_3	^{t}Bu	PNP	-Mn-N, +Mn-C, -P-C, +P-O, +Mn-O
122	OH-PNP-CF_3-2	6	-10.84	OH	CF_3	H	PNP	+P-O
123	OH-PNP-CF_3-15	5	-3.58	OH	CF_3	H	PNP	-Mn-N, +Mn-C, +N-H, -P-C, +P-O, -O-H, +Mn-O
124	OH-PNP-CF_3-21	5	-1.55	OH	CF_3	H	PNP	-Mn-N, -Mn-P, +Mn-C, +N-H, -P-C, +P-O, -O-H
125	OH-PNP-cy-^{t}Bu-2	5	-71.34	OH	cy	^{t}Bu	PNP	-Mn-N
126	OH-PNP-^{i}Pr-^{t}Bu-2	5	-70.23	OH	^{i}Pr	^{t}Bu	PNP	-Mn-N
127	Br-SNS-H-cy-2	6	-3.41	Br	H	cy	SNS	-Mn-S, +Mn-H
128	Br-SNS-H-cy-3	6	-2.68	Br	H	cy	SNS	-Mn-S, +Mn-H
129	Br-SNS-H-cy-5	6	-10.68	Br	H	cy	SNS	-Mn-S, +Mn-H
130	OH-SNS-CF_3-cy-5	5	-8.59	OH	CF_3	cy	SNS	-Mn-S, -Mn-S, -Mn-N, +Mn-F, +Mn-F, +H-O, +N-C, -C-F, -C-F
131	OH-SNS-H-ph-3	5	-9.48	OH	H	ph	SNS	-Mn-S, -H-S, +H-O

References

(1) Bougueroua, S.; Spezia, R.; Pezzotti, S.; Vial, S.; Quessette, F.; Barth, D.; Gaigeot, M.-P. Graph theory for automatic structural recognition in molecular dynamics simulations. *The Journal of Chemical Physics* **2018**, *149*, 184102.

(2) McKay, B. D.; Piperno, A. Practical graph isomorphism, II. *Journal of symbolic computation* **2014**, *60*, 94–112.

(3) Hashemi, A.; Bougueroua, S.; Gaigeot, M.-P.; Pidko, E. A. ReNeGate: A Reaction Network Graph-Theoretical Tool for Automated Mechanistic Studies in Computational Homogeneous Catalysis. *J. Chem. Theory Comput.* **2022**, *18*, 7470–7482.
